# *Setd5* haploinsufficiency alters neuronal network connectivity and leads to autistic-like behaviors in mice

**DOI:** 10.1038/s41398-018-0344-y

**Published:** 2019-01-17

**Authors:** Spencer M. Moore, Jason S. Seidman, Jacob Ellegood, Richard Gao, Alex Savchenko, Ty D. Troutman, Yohei Abe, Josh Stender, Daehoon Lee, Sicong Wang, Bradley Voytek, Jason P. Lerch, Hoonkyo Suh, Christopher K Glass, Alysson R. Muotri

**Affiliations:** 1 Biomedical Sciences Graduate Program, University of California, San Diego, La Jolla, CA USA; 2 Department of Pediatrics, School of Medicine, University of California, San Diego, La Jolla, CA USA; 3 Department of Cellular and Molecular Medicine, School of Medicine, Universityof California, San Diego, La Jolla, CA USA; 4 Mouse Imaging Centre, Hospital for Sick Children, Toronto, ON Canada; 5 Neurosciences and Mental Health Program, Hospital for Sick Children, Toronto, ON Canada; 6 Department of Cognitive Science, University of California, San Diego, La Jolla, CA USA; 7 Department of Stem Cell Biology and Regenerative Medicine, Lerner Research Institute, Cleveland Clinic, Cleveland, OH USA; 8 Neurosciences Graduate Program, University of California, San Diego, La Jolla, CA USA; 9 5Halıcıoğlu Data Science Institute, University of California, San Diego, La Jolla, CA USA; 10 Kavli Institute for Brain and Mind, La Jolla, CA USA; 11 Department of Medical Biophysics, University of Toronto, Toronto, ON Canada; 12 Rady Children’s Hospital San Diego, San Diego, CA USA; 13 Stem Cell Program, University of California, San Diego, La Jolla, CA USA; 14 Center for Academic Research and Training in Anthropogeny (CARTA), University of California, San Diego, La Jolla, CA USA

## Abstract

*SETD5*, a gene linked to intellectual disability (ID) and autism spectrum disorder (ASD), is a member of the SET-domain family and encodes a putative histone methyltransferase (HMT). To date, the mechanism by which *SETD5* haploinsufficiency causes ASD/ID remains an unanswered question. *Setd5* is the highly conserved mouse homolog, and although the *Setd5* null mouse is embryonic lethal, the heterozygote is viable. Morphological tracing and multielectrode array was used on cultured cortical neurons. MRI was conducted of adult mouse brains and immunohistochemistry of juvenile mouse brains. RNA-Seq was used to investigate gene expression in the developing cortex. Behavioral assays were conducted on adult mice. *Setd5*^+/−^ cortical neurons displayed significantly reduced synaptic density and neuritic outgrowth in vitro, with corresponding decreases in network activity and synchrony by electrophysiology. A specific subpopulation of fetal *Setd5*^+/−^ cortical neurons showed altered gene expression of neurodevelopment-related genes. *Setd5*^+/−^ animals manifested several autism-like behaviors, including hyperactivity, cognitive deficit, and altered social interactions. Anatomical differences were observed in *Setd5*^+/−^ adult brains, accompanied by a deficit of deep-layer cortical neurons in the developing brain. Our data converge on a picture of abnormal neurodevelopment driven by *Setd5* haploinsufficiency, consistent with a highly penetrant risk factor.

## Introduction

The SET domain-containing family of HMTs encompasses the largest group of writers to the histone methylation code and is characterized by an evolutionarily conserved 130 amino acid SET domain. Human *SETD5*, located on chromosome 3p, stretches 23 exons in its full transcript and encodes the SET domain-containing protein 5, a 1442 amino acid protein. Its primary structure is highly conserved across vertebrates. Its role in development in general, and CNS development specifically, is increasingly appreciated. In 2012, the Jenuwein group purified SETD5 from HeLa extracts from a protocol to isolate HMTs, and recombinant mouse Setd5 was found to have H3K9 HMT activity^1^. Osipovich et al. found that mouse Setd5 co-immunoprecipitated with the NCoR complex of transcriptional regulators, and that *Setd5*^*−/*−^ homozygous knockout animals were embryonic lethal beyond E11, due to multiple developmental defects including failure of neural tube closure and cardiac abnormalities^2^. Not only does SETD5 exert influence over critical elements of early development, but also it has been implicated in human neurodevelopmental disorders.

Converging evidence suggests that loss-of-function (LoF) mutations in *SETD5* cause neurodevelopmental defects in humans^3^. *SETD5* emerged as a candidate from bioinformatics analysis of a cohort of 996 patients in the United Kingdom with ID. Researchers found overlapping phenotypes in ID patients harboring heterozygous *SETD5* LoF mutations and the existing 3p microdeletion syndrome, which spans *SETD5* on the short arm of chromosome 3^4^. A subsequent study performing whole exome sequencing (WES) from a cohort of 250 ID patients found seven patients with missense, nonsense, splice-site, or microdeletion *SETD5* mutations^5^. This study attributed the microdeletion patients’ phenotype to nonsense-mediated decay (NMD) based on reduced *SETD5* mRNA transcript levels in HEK293 cells transfected with CRISPR/Cas9 guide-RNAs (gRNA) recapitulating the patients’ microdeletion^5^. Pinto et al. identified *SETD5* as a candidate autism spectrum disorders (ASD) susceptibility gene based on the discovery of de novo copy number variants (CNVs) observed in a cohort of 2446 ASD patient genomes^6^.

We took advantage of the *Setd5*^+/−^ heterozygous mouse as a model of Setd5 LoF. We hypothesized that LoF would be particularly pronounced in neurons from cerebral cortex, which is widely implicated in ASD pathogenesis^7^ and a cell type enriched in *SETD5/Setd5* expression^8^. To elucidate the role of Setd5 in nervous system development and its contributions to ASD/ID, we studied the morphological and electrophysiological features of dissociated cortical neurons, the molecular and transcriptomic signature underlying Setd5 reduction, neuroanatomical details of adult and developing brains, and finally, behavioral phenotypes of adult animals.

## Materials and Methods

### Primary cortical neuron culture

Primary cortical neurons were harvested from E18.5 or P0 animals as described previously^9^ and grown in vitro on glass. Ara-C was used to arrest proliferation of glial progenitors in neuronal cultures.

### Immunohistochemistry/immunocytochemistry

Mouse brain tissue for immunohistochemistry was collected by perfusion-fixation and frozen sections. Sections were permeabilized, blocked, and incubated in primary antibody solution overnight (Table 1), followed by a fluorescently-labeled secondary antibody and nuclei staining. For cells, they were fixed, permeabilized, blocked then incubated in primary antibody solutions overnight, followed by a fluorescently-labeled secondary antibody and nuclei staining. Neuron morphology was assessed by Neurolucida tracing. Synapses were quantified by the co-localization of pre- and post-synaptic puncta as previously described^10^. Cell death was quantified by TUNEL staining. For all assays, unstained controls were used to ensure no background GFP staining was observed in *Setd5*^+/−^ samples.

**Table 1:** List of all antibodies used in the study

Application	Antibody	Dilution	Manufacturer	Catalog
Immunocytochemistry	MAP2	1:2000	Abcam	5392
	CTIP2	1:500	Abcam	25B6
	VGLUT1	1:500	Synaptic systems	135011
	HOMER1	1:500	Synaptic systems	160003
	GFAP	1:1000	Dako	Z0334
	NEUN	1:1000	Millipore	377
	GAD65/67	1:1000	Abcam	11070
	GFP	1:1000	Abcam	290
Immunohistochemistry	BRN2	1:500	Santa Cruz	6029
	TBR1	1:500	Abcam	31940
	CTIP2	1:500	Abcam	25B6
	PSD95	1:500	Abcam	18258
	SATB2	1:500	Abcam	51502
Flow cytometry	CD24-PE	1:3000	Abcam	218742
	CD45-AF647	1:600	Biolegend	103124

### Electrophysiology

Primary cortical neurons were cultured on Axion Maestro multielectrode array (MEA) plates and spontaneous spike activity recorded serially starting at DIV. LFP computation was obtained by a custom MATLAB algorithm to calculate the power ratio at 1–10 and 100–150 Hz ranges. For imaging of calcium transient activity, cells were loaded with the calcium-sensing dye Fluo-4AM and spontaneous firing activity was recorded.

### Electroencephalogram

First, spontaneous EEG activity was recorded in awake behaving adult mice via hippocampal-implanted electrodes. Next, seizure threshold was assessed by measuring latency to epileptic spikes provoked by the convulsant PTZ.

### RNA-Seq/scRNA-Seq

Primary cortical neurons from E18.5 fetuses were extracted as described^11^. Once in single-cell suspension, they were stained with pan-neuronal marker CD24 and hematopoietic marker CD45 and sorted by fluorescence-activated cell sorting (FACS). Live neuronal cells were collected and RNA libraries prepared and sent for bulk RNA-Seq. Libraries were sequenced and gene ontology (GO) analysis conducted of differentially expressed genes. For single-cell (sc) RNA-Seq, the same sorting protocol was used, but cells were then used to create single-cell libraries prior to sequencing. t-SNE plots were generated and cells clustered. The most highly expressed genes and differentially expressed genes within the clusters were analyzed.

### Animals

The *Setd5*^GFP^ transgenic mouse^2^, hereafter referred to as the *Setd5*^+/−^ heterozygous knockout animal was used for all animal experiments in accordance with the Institutional Animal Care & Use Committee (IACUC) at UCSD and Cleveland Clinic.

### Neurologic and metabolic assays

Weight, grip strength, rotorod^12^, and composite neurological score^13^ were measured at age 10 weeks.

### Behavioral assays

Behavioral experiments were conducted 1 week apart to prevent test fatigue, using two independent cohorts. The open field test was conducted as described previously^12^. The Barnes maze was conducted as described previously^14^. The elevated plus maze was conducted as described previously^15^. Overnight nest building was performed as described previously^14^.

#### 3-chamber social interaction

The 3-chamber test was conducted as described previously^14,16^ in three subtrials: sociability (novel animal vs. empty), social novelty (novel animal vs. familiar), and social preference (cagemate animal vs. novel).

### Magnetic resonance imaging

Brain MRI was performed of fixed adult mouse skulls perfused with gadolinium-containing contrast aent as previously described^17^. The volume of various brain regions was assessed and compared by genotype.

### Microscopy

Fluorescent microscope images were acquired on a Zeiss Apotome microscope using Zen software.

### Statistics

Detailed statistical methods are included in the description of individual experiments. Statistical analysis was conducted with GraphPad Prism 7 software, generally by *t-*test or 2-way ANOVA with *α* = 0.05. A false discovery rate (FDR) significance value of 0.05 was used to for tests with multiple comparisons.

## Results

### Morphological alterations and hypoconnectivity in *Setd5*^+/−^ primary cortical neurons

**Fig. 1:**
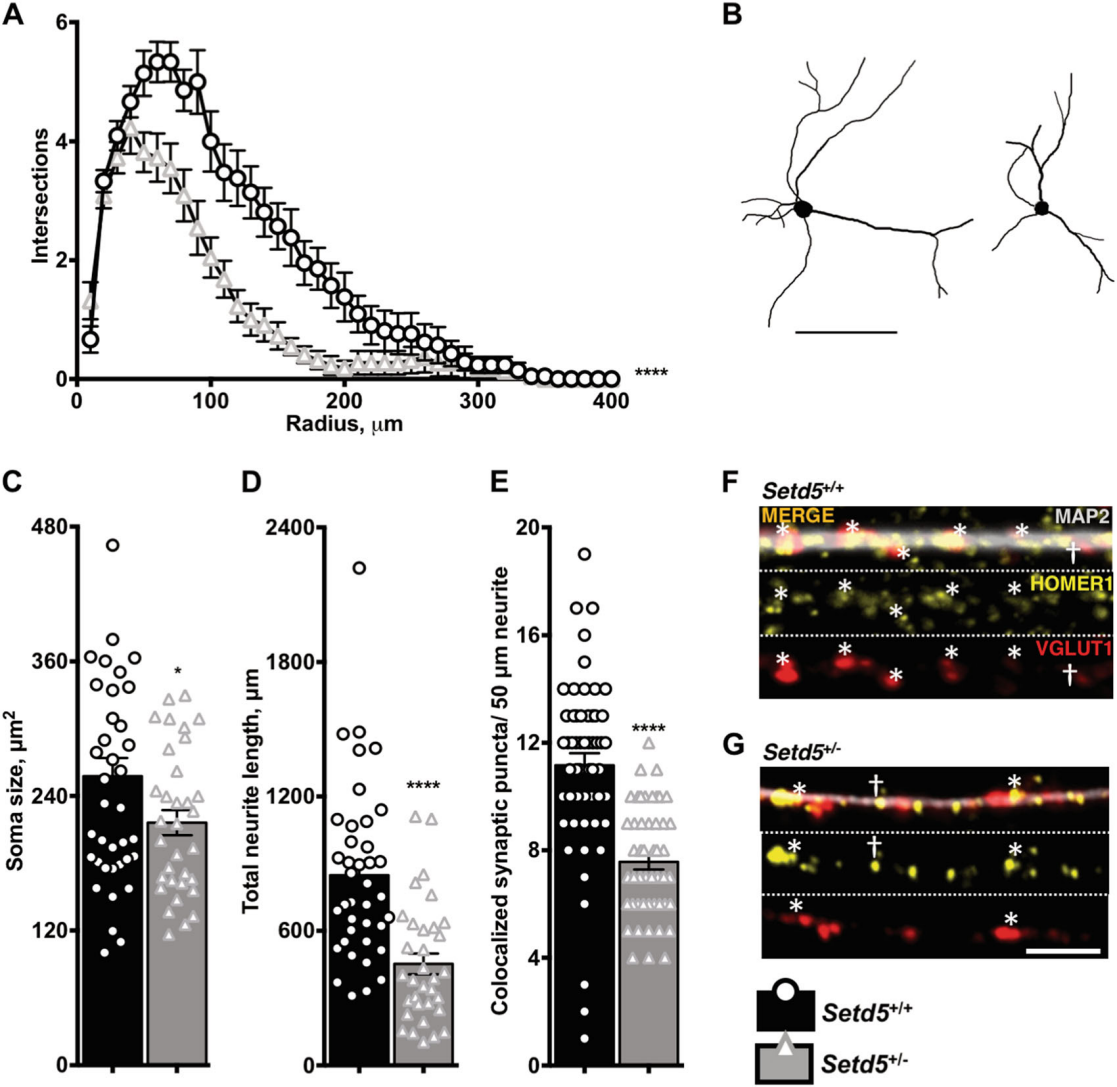
*Setd5*^+/−^ primary cortical neurons display signs of morphological alterations consistent with hypoconnectivity. **a** Het neurons present significantly fewer intersections by Sholl analysis (genotype ****P < 10^−4^, F_1,1640_ = 169.8, DF = 1). **b** Representative traces of wt (left) and het (right) neurons are depicted (scale bar 100 μm). **c** Het neurons have significantly smaller soma (*P = 0.0461, *t*_1,70_ = 2.031, DF = 70). **d** Total neuritic outgrowth is significantly reduced among het neurons (****P < 10^−4^, *t*_1,70_ = 4.94, DF = 70). **e** HOMER1^+^ VGLUT1^+^ colocalized synaptic puncta are significantly less dense in MAP2^+^ neurites of het neurons (****P < 10^−4^ *t*_1,104_ = 6.643, DF = 104). **f**–**g** Representative image of wt and het neurite synaptic puncta quantification; top = merge MAP2 (white), VGLUT1 (red), HOMER1 (yellow); center = VGLUT1 (red); bottom = HOMER1 (yellow); *asterisks represent colocalized synaptic puncta, while ^†^dagger represents uncounted non- colocalized punctum, scale bar 5 μm. Statistics: *n* = neurons from 12 total animals per genotype from three total experiments. (**a**–**d**) = 39 *Setd5*^*+/+*^ and 33 *Setd5*^*+/−*^ CTIP2^+^ neurons, with group means compared by *t*-test (**b**–**d**) or 2-way ANOVA (**a**) for factors radius and genotype. (**f**) = 55 *Setd5*^*+/+*^ and 51 *Setd5*^+/−^ neurons, with group means compared by *t*-test. Replicates as individual neurons (**a**–**e**) with error bars representing mean ± SEM

Alterations in neuronal morphology and function were previously described for other ASD-related chromatin regulator genes^9^. Thus, we cultured primary cortical neurons harvested from P0 *Setd5*^+/+^ and *Setd5*^+/−^ animals in vitro for 24 days. Ctip2^+^ cells were selected for analysis as the predominant projection neurons in the cultures with extensive measurable arborization. WT (left) and het (right) neurons are depicted (Fig. 1b, scale bar 100 μm); when subjected to Sholl analysis, het neurons were found to have significantly fewer intersections with 10 μm concentric circles (F_1,1640_ = 169.8, *P* < 10^−4^, Fig. 1a). *Setd5*^+/−^ neurons also had significantly smaller soma size (257.7 ± 16.2 vs. 216.2 ± 11.19 μm^2^, *P* = 0.0461, Fig. 1c) and significantly reduced neuritic outgrowth (848.1 ± 62.41 vs. 452.2 ± 46.27 μm, *P* < 10^−4^, Fig. 1d) despite no significant differences in total neurite number (3.282 ± 0.127 vs. 3.091 ± 0.1649, *P* = 0.3545, data not shown). When stained by immunocytochemistry for the pre- and post-synaptic markers VGLUT1 and HOMER1, respectively, *Setd5*^+/−^ neurons had reduced density of colocalized pre- and post-synaptic puncta on MAP2^+^ neurites (11.16 ± 0.4508 vs. 7.569 ± 0.2818 puncta/50 μm, *P* < 10^−4^, Fig. 1e–g, scale bar 5 μm). To determine whether differences in cell viability contributed to the observed morphological differences, fixed cells were subject to terminal deoxynucleotidyl transferase dUTP nick end labeling (TUNEL). No difference in percent apoptotic neurons was detected (14.57 ± 2.895 vs. 10.21 ± 2.254 percent, *P* = 0.3220, Fig. S1A). To determine whether *Setd5* LoF impacted excitatory:inhibitory balance, a purported aspect of aberrant network connectivity in ASD^18^, fixed cultures were stained for the excitatory and inhibitory markers VGLUT1 and GAD65/67, respectively. No differences in percent VGLUT1^+^ cells (77.09 ± 4.389 vs. 70.87 ± 3.457 percent, *P* = 0.2821, Fig. S1B) or percent GAD65/67^+^ cells (32.5 ± 4.22 vs. 38.52 ± 3.154 percent, *P* = 0.2700, Fig. S1B) were detected. To exclude any effects of culture heterogeneity on morphology and synaptogenesis observed in neurons, cell cultures were fixed and stained for the mature postmitotic neuron marker NeuN and glial marker GFAP, in which no differences in percent neurons (82.55 ± 3.10 vs. 80.72 ± 4.04 percent, *P* = 0.7181, Fig. S1C) or glia (17.45 ± 3.10 vs. 19.28 ± 4.04 percent, *P* = 0.7181, Fig. S1C) were observed. As a further inquiry into culture heterogeneity, no genotype-dependent difference in percent positive CTIP2^+^ neurons was detected (48.43 ± 3.40 vs. 51.60 ± 4.11 percent, *P* *=* 0.5571, Fig. S1D).

### Delayed network development in *Setd5*^+/−^ cortical neurons

**Fig. 2:**
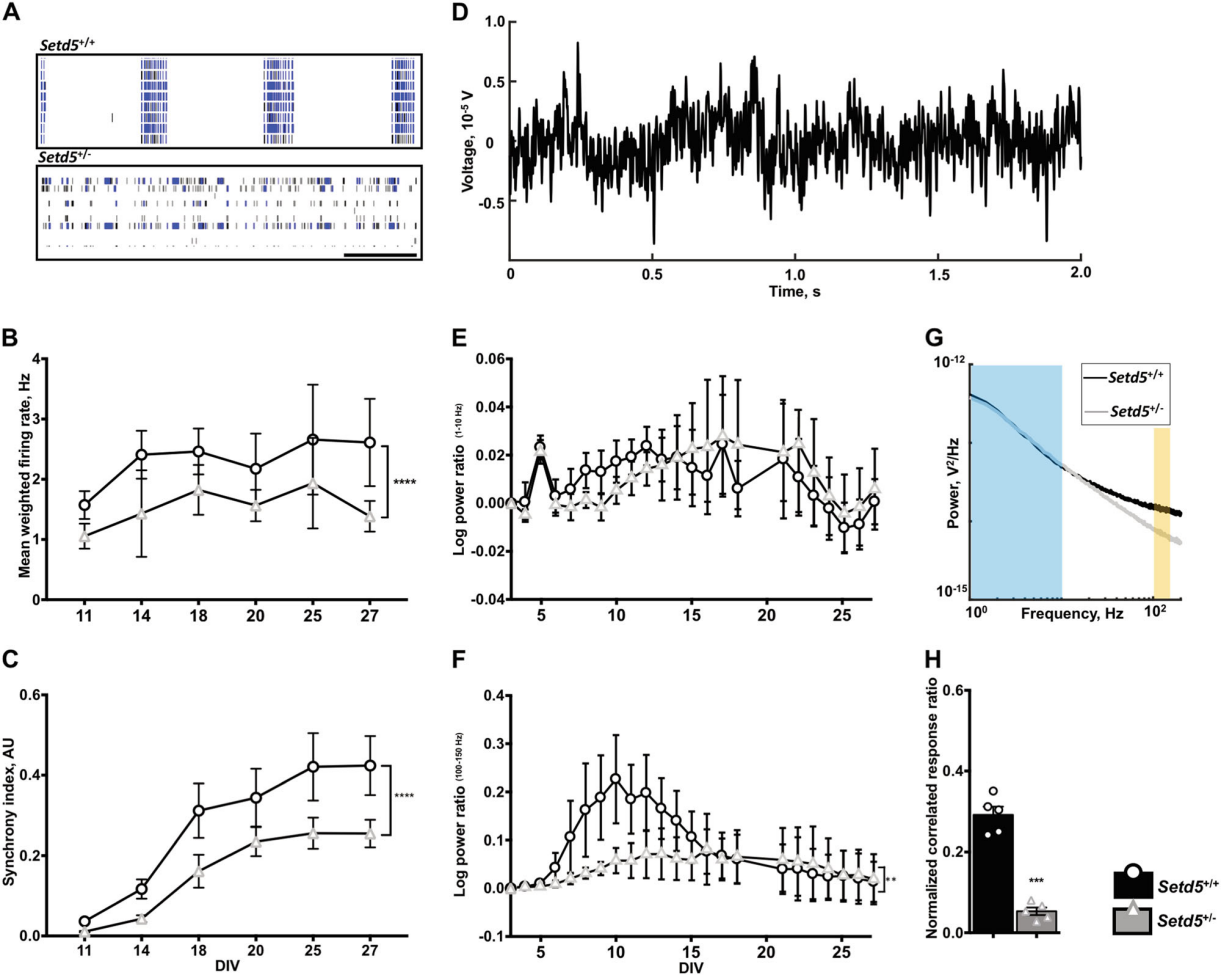
*Setd5*^+/−^ cortical neurons trail wt counterparts in development of synchronized neural networks. **a** Raster plots depict spontaneous firing activity in eight electrodes from a single well (wt, top; het, bottom) showing a qualitatively different firing pattern (scale bar 20 s). **b** Spontaneous spike activity is significantly reduced in het cells (genotype ****P < 10^−4^, F_1,341_ = 22.37, DF = 1). **c** Synchrony index among het neurons lags significantly behind wt (genotype ***P < 10^−3^, F_1,379_ = 12.86, DF = 1). **d** Example LFP trace after low-pass filtering raw MEA signal at 500 Hz. **e**, **f** Log_10_ power ratio baselined to first day of MEA recording for low-frequency (1–10 Hz, **e**) and high-frequency/broadband gamma (100–150 Hz, **f**) power, with genotype-dependent differences in power ratio for high-frequency only [(entire time period: low-frequency F_1,45_ = 0.55, *P* = 0.4644 and high-frequency F_1,45_ = 10.17, **P = 0.0042); (6–11 DIV only: low-frequency F_1,11_ = 18.91, **P = 0.0074 and high-frequency F_1,11_ = 33.36, **P = 0.0022)]. **g** Example power spectral densities (PSD) from Setd5^+/+^ (black) and Setd5^+/−^ (gray) cultures. Blue and yellow boxes denote low- and high-frequency regions of interest for (**e**) and (**f**), respectively. **h** Significantly reduced correlated firing activity among het neurons (****P < 10^−4^, *t*_1,12_ = 10.65, DF = 8) measured by Ca^2+^ transients. Statistics: (**b**, **c**) *n* = neurons from 12 *Setd5*^+/+^ and *Setd5*^+/−^ animals each; 2-way ANOVAs for factors genotype and DIV; replicates = individual wells (neurons from 1 animal/well) pooled from three separate experiments. (**e**, **f**) Replicates defined as data from neurons of individual animals, *n* = 8 Setd5^+/+^ and *n* = 4 Setd5^+/−^; 2-way ANOVAs for factors genotype and DIV. **h** *n* = neurons from five animals per genotype (583 *Setd5*^+/+^ or 505 *Setd5*^+/−^ active total neurons), with each animal as a single replicate and *t-*test of group means. Error bars representing mean ± SEM

To ascertain the functional consequences of altered morphology and hypoconnectivity among *Setd5*^+/−^ cortical neurons and investigate a potential cellular basis for epileptogenesis in *SETD5* human patients, cells were cultured atop a multielectrode array (MEA), with serial recordings obtained beginning at 8 days in vitro (DIV, Table 2). Representative raster plots of spontaneous spike activity from 21 DIV are depicted (Fig. 2a, scale bar 20 s). When spontaneous neural recordings were analyzed, *Setd5*^+/−^ neurons had significantly reduced weighted mean firing rate (*P* < 10^−4^, Fig. 2b). Bursting activity also had genotype-dependent differences: *Setd5*^+/−^ neurons had significantly reduced normalized burst frequency (*P* = 0.0002, data not shown), with bursts spreading to significantly fewer electrodes (*P* < 10^−4^, data not shown). Moreover, *Setd5*^*+/−*^ neuron firing was significantly less synchronous (*P* = 0.0004, Fig. 2c). To ensure spiking originated from neuronal-initiated action potential firing, 10 nM TTX was added to the cultures, which completely abolished spiking (data not shown). Complementary to the spiking results, local field potential (LFP) recordings obtained from raw MEA data (Fig. 2d) showed genotype-dependent low- and high-frequency power changes (Fig. 2g), providing evidence for network-level differences in activity. Low-frequency (1–10 Hz) transients in the LFP—markers of network synchronized depolarization^19^—were not significantly different between genotypes overall, although intervals of difference did emerge, especially within the second week in culture (6–11 DIV, Fig. 2e). Interestingly, high-frequency (100–150 Hz) power—sometimes known as broadband or high gamma power, an index of local circuit firing rate mostly provided by neuronal activity^20^—was significantly different between genotypes (*P* = 0.0042, Fig. 2f), with particularly pronounced differences in the 6–11 DIV critical period.

**Table 2:** Recording parameters for multielectrode array

Axion maestro multielectrode array recording protocol	
Configuration	Real-time spontaneous neural
Length	30 min
Spike activity criteria	Field potential ≥ 6*σ* above noise
Active electrode criteria	5 spikes•min^−1^
Burst criteria	Spikes in ≥ 5 active electrodes

Quality controls to ensure culture heterogeneity did not contribute to network-level electrophysiological differences were conducted. While cell viability was shown to be comparable between genotypes (Fig. S1A), to ensure that variation in cell density did not contribute to network connectivity, cells were imaged and observed at comparable coverage of the MEA (Fig. S1E, F), and total protein collected from each well was comparable between genotypes (*P* = 0.6142, data not shown).

While MEA analysis provides a global overview of network connectivity, it lacks single-cell optical resolution afforded by other modalities. Calcium signaling in neurons mediates several critical functions including synaptic plasticity, and calcium channelopathies have been implicated in select monogenic neurodevelopmental conditions such as Timothy syndrome^21,22^. To determine whether Setd5 haploinsufficiency impacted calcium signaling in cortical neurons, we employed optical recordings using a fluorescent dye to monitor calcium transients (Fig. S2A, B, scale bar 50 μm) and quantified the neuronal traces (three representative traces, Fig. S2C). Neurons from *Setd5*^+/−^ cultures demonstrated reduced synchrony of spontaneous transients, with significantly fewer neurons participating in correlated firing activity (0.292 ± 0.0454 vs. 0.053 ± 0.021 correlated firing response ratio, *P* < 10^−4^, Fig. 2h).

### Developing brain single-cell transcriptomic analysis

**Fig. 3:**
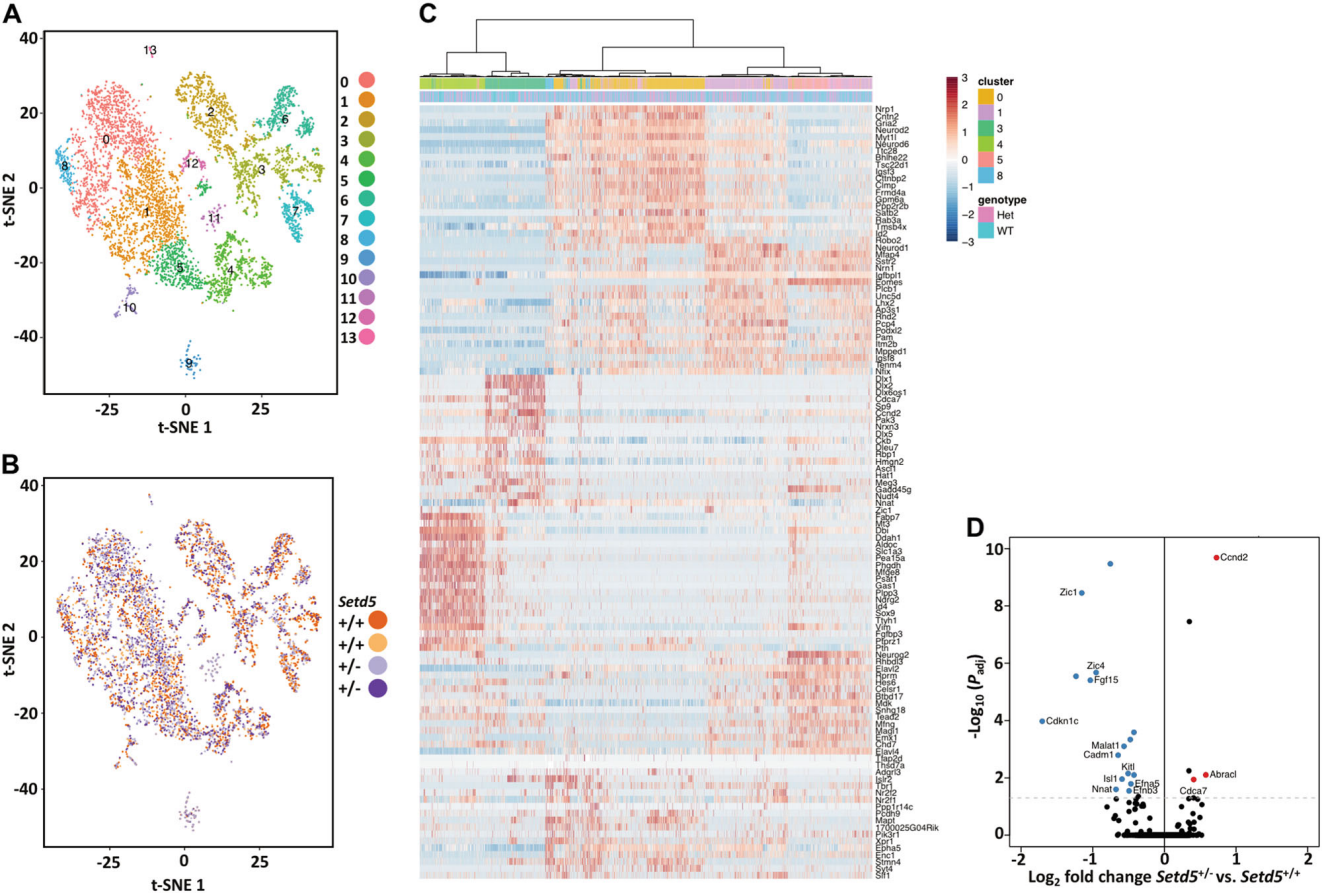
Single-cell transcriptomic profiling of E18.5 *Setd5*^+/−^ cortical progenitors. **a**, **b** Whale-octopus plots of t-distributed stochastic neighbor embedding (t-SNE) dimensional reduction of barcoded single-cell RNA-Seq reads into 14 clusters (**a**) distributed across both genotypes (**b**). **c** Hierarchical clustering of Z-scaled single-cell expression values reveals separation of t-SNE clusters and distribution of genotypes within individual clusters. **d** Volcano plot of cluster 3 genes showing differentially expressed genes in *Setd5*^+/−^ (significantly downregulated: blue; significantly upregulated: red; select genes of interest labeled) Statistics: Sorted CD24^+^ CD45^−^ cortical suspensions from *n* = 2 E18.5 fetuses per genotype. *n* = 2066–2858 cells, 39805–54534 reads/cell (*Setd5*^+/+^) and 2269–2645 cells, 48174–56267 reads/cell (*Setd5*^+/−^)

We next focused on potential molecular differences in the neurons from the mouse brain. We performed bulk RNA-Seq of sorted neuronal cells (CD24^+^, CD45^−^, Fig. S3) from the developing (E18.5) cortex. Gene ontology analysis of differentially expressed (DE) genes (82 total with FDR *P* < 0.05) revealed 20 ontological categories (Fig. S4). Although extracellular matrix-related genes were significantly downregulated in *Setd5*^+/−^ cells, this did not fully reveal a transcriptomic basis for the morphological and electrophysiological differences we observed in *Setd5*^+/−^ neurons (Supplemental Sheet 1). We repeated the dissection and sorting, then used single-cell (sc) RNA-Seq to reveal potential genotype-dependent differences in the developing cortex cellular composition. Pooled data from both genotypes was clustered using the Seurat R package^23^, and the resulting 14 clusters were displayed using t-distributed stochastic neighbor embedding (t-SNE, Fig. 3a, b). The most highly expressed genes in each cluster are listed (Supplemental sheet 2). Clusters 0, 1, 3, 4, 5, and 8 were identified for more extensive heatmap analysis (Fig. 3c) based on enrichment of neurodevelopmental genes among the top 20 expressed genes. Hierarchical clustering separated these six clusters (Fig. 3b). Cluster 3 had the greatest number of significantly (FDR *P* < 0.05) DE genes by genotype. Literature search of Cluster 3 DE genes revealed effects of *Setd5* haploinsufficiency in upregulation (*Ccnd2*, *P* < 10^−9^; *Abracl*, *P* = 0.0080; *Nr2f1*, *P* = 0.0155; *Cdca7*, *P* = 0.0503) or downregulation (*Zic1, P* < 10^−8^; *Zic4*, *P* < 10^−5^; *Fgf15, P* < 10^−5^; *Malat1*, *P* = 0.0008; *Cadm1*, *P* = 0.0016; *Pnoc*, *P* = 0.0080; *Kitl, P* = 0.0084; *Efna5*, *P* = 0.0161; *Nnat*, *P* = 0.0318; *P* = 0.0290, Fig. 3d, Supplemental Sheet 1) of select genes related to nervous system development. Relative expression by cluster and genotype of select neural marker genes is also depicted (heatmap, Fig. S5A, split-dot plot, Fig. S5B).

### *Setd5*^+/−^ animals have abnormal patterns of social interaction and demonstrate autism-compatible behaviors

**Fig. 4:**
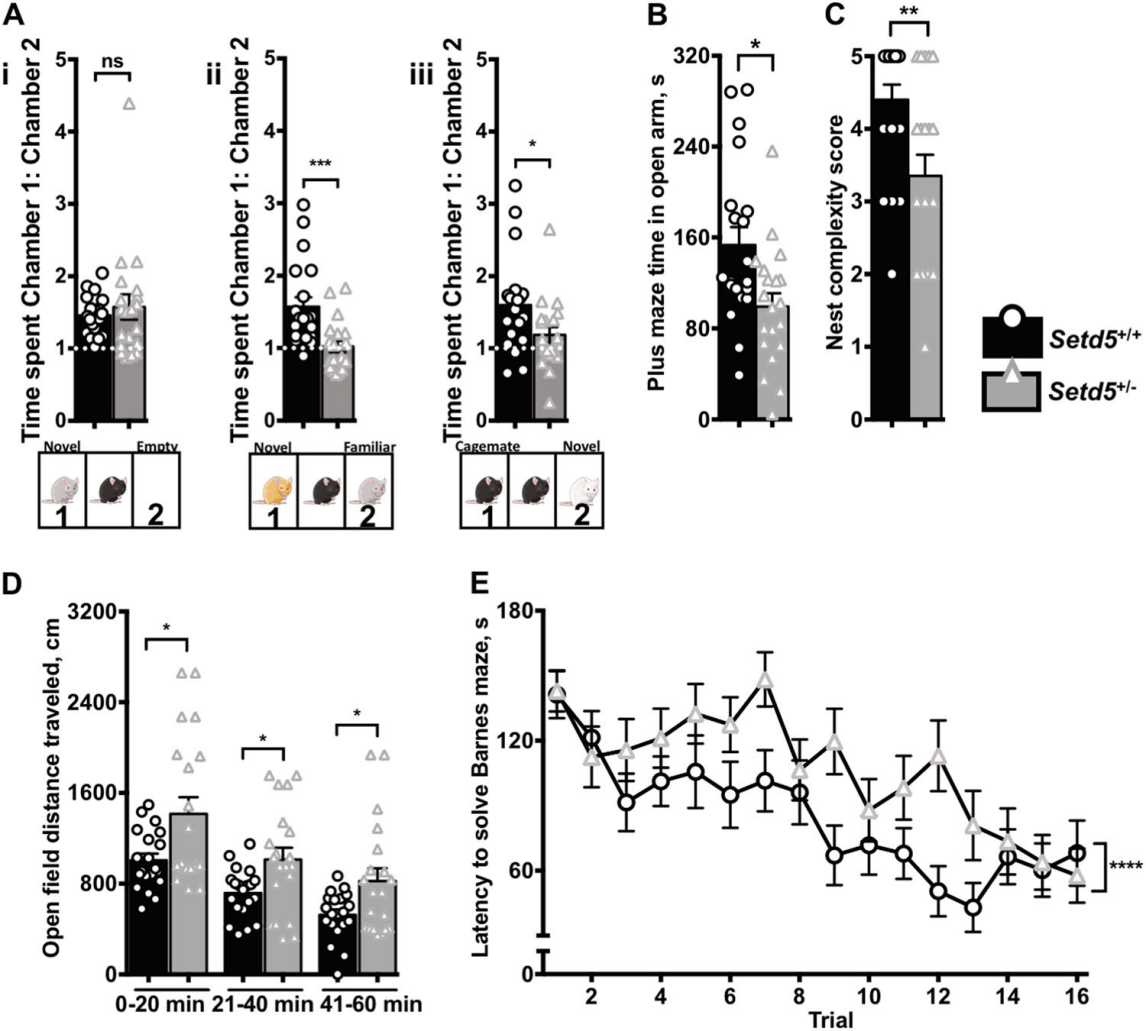
*Setd5*^+/−^ animals display autism-compatible behaviors. Pooled male-female data of behavioral tasks illustrating genotype-dependent differences. **a** (i) 3-chamber social interaction test. Left: No genotype-dependent differences in social approach as measured by ratio of time spent in chamber with novel animal vs. empty chamber (1.457 ± 0.060 vs. 1.575 ± 0.175, *P* = 0.5274, *t*_1,38_ = 0.6379). (ii) Center: Het mice lack normal preference for novel over familiar animal as measured by ratio of time spent in chamber with novel animal vs. familiar animal (1.573 ± 0.130 vs. 1.021 ± 0.078, **P = 0.008, *t*_1,38_ = 3.643). (iii) Het mice lack normal preference for cagemate over novel animal as measured by ratio of time spent in chamber with cagemate animal vs. novel animal (1.599 ± 0.149 vs. 1.187 ± 0.108, *P = 0.031, *t*_1,38_ = 2.24). **b** Het mice spend significantly less time in open arms of elevated plus maze (*P = 0.0112, F_1,36_ = 7.146, DF = 1). **c** Het mice build significantly less complex nests in overnight nest-building task (genotype **P = 0.0074, Mann–Whitney ranked U = 105.5, DF = 1). **d** Het mice are hyperactive in open field (genotype ****P < 10^−4^, F_1,2_ = 18.27, DF = 1). **e** Het mice are impaired on Barnes maze (genotype ****P < 10^−4^, F_1,15_ = 21.8, DF = 21.8). Statistics: *n* = 10 total animals per sex, per genotype; *t-*test of pooled male-female data (**a**, **b**, **d**); Mann–Whitney ranked test (**c**); 2-way ANOVA (**e**). Graphs are representative of pooled male and female data for each genotype, with replicates as individual animals and error bars mean ± SEM

We subjected independent cohorts of adult animals to established behavioral assays in animal models of ASD^15,24^. The 3-chamber social interaction test captures differences in the experimental animal’s preference for interacting with a known versus novel animal and is frequently employed in animal models of autism. Although *Setd5*^+/−^ animals had no defects in social approach compared with controls (1.457 ± 0.060 vs. 1.575 ± 0.175 ratio of time with novel mouse vs. empty chamber, *P* = 0.5274, *t*_1,38_ = 0.6379, Fig. 4ai), they lacked the normal preference for novel over familiar (1.573 ± 0.130 vs. 1.021 ± 0.078, ratio of time with novel mouse vs. familiar mouse, *P* = 0.008, *t*_1,38_ = 3.643, Fig. 4aii) in the social novelty preference test. This trend was repeated in the test of social preference for cagemate versus novel mouse (1.599 ± 0.149 vs. 1.187 ± 0.108, ratio of time time with cagemate mouse vs. novel mouse, *P* = 0.031, *t*_1,38_ = 2.24, Fig. 4aiii), suggesting that het animals failed to distinguish known from unknown conspecifics. *Setd5*^+/−^ mice spent significantly reduced time in the open arms of the elevated plus maze versus wt controls (153.3 ± 15.91 vs. 99.25 ± 11.81 s, *P* = 0.0096, Fig. 4b), suggestive of increased anxiety. They also built significantly less complex nests in the overnight nest-building task (nest scores 4.4 ± 0.2103 vs. 3.35 ± 0.2927, *P* = 0.0074, Fig. 4c). We also conducted an open field test, finding significantly elevated activity levels among *Setd5*^+/−^ animals over the course of the 1 h test (*t*-test *P* values 0.0135 0–20 min; 0.0148 21–40 min; 0.0175 41–60 min; ANOVA genotype *P* < 10^−4^, Fig. 4d). Thigmotaxis, the tendency of rodents to prefer the perimeter of the open field task, was also affected: genotype was found to be a significant factor accounting for variation in time spent in the central 25% of the chamber (genotype *P = 0.0343, Table 3). Finally, the Barnes maze task of cognition and spatial memory and recall was conducted: *Setd5*^+/−^ animals displayed significantly reduced performance over the course of the 16 trials versus wt controls (*P* < 10^−4^, Fig. 4e). Complete data when separated by sex (Fig. S6) and analyzed by ANOVA (Table 3) are displayed. Gross neurological deficits in the het animals were ruled out by the observation of comparable performance on rotorod, grip strength, and composite neurological tests^13,25^ (Fig. S7).

**Table 3:** Complete statistics for behavioral experiments

Behavioral task	Genotype *P*	Genotype F	Sex *P*	Sex F
Sociability	0.5317	0.3989	0.2909	1.149
Social-novelty preference	0.004	15.06	0.0131	6.821
Social-preference	0.0299	5.1112	0.384	0.7768
Elevated plus maze	0.0112	7.146	0.91	0.013
Nest building	0.0071	8.150	0.8296	0.0185
Barnes maze	<0.0001	21.80	0.0002	13.65
Open field	<0.0001	18.27	0.3495	0.8829
Open field time in center	0.0343	4.891	0.0103	7.433

Epileptic activity is a known feature of certain *SETD5* LoF patients^3^. Thus we subjected adult mice to electroencephalogram (EEG) via hippocampal-implanted electrodes to determine whether the animal model recapitulated patient seizures. Neither *Setd5*^+/+^ nor *Setd5*^+/−^ animals showed spontaneous seizure activity (Fig. S8A, B), and no genotype-dependent differences in seizure threshold were detected when sub-convulsive doses of PTZ (pentylenetetrazol) were successively injected prior to recording (*P* *=* 0.6549, Fig. S8C–F).

### Anatomical changes in adult mouse brain by MRI

**Fig. 5:**
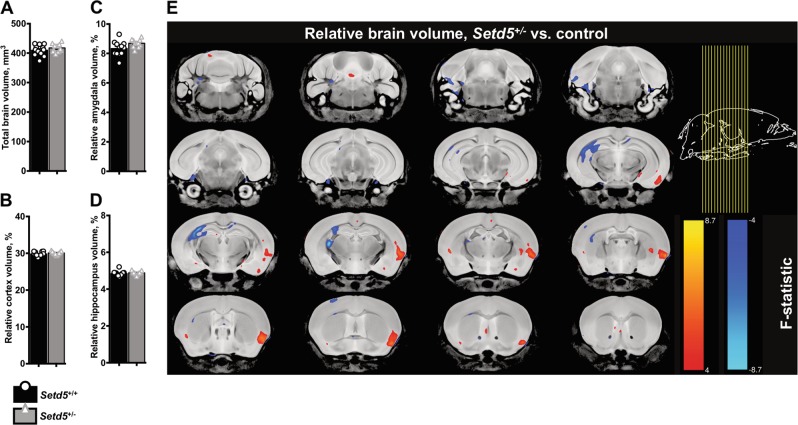
Anatomical differences in brain region volume in Setd5^+/−^ adults. **a** No genotype-dependent difference in brain volume (409.7 ± 5.29 vs. 417.5 ± 4.16 mm^3^, *t*_1,21_ = 1.156, *P* = 0.2606). **b** No genotype-dependent difference in relative cortex volume (29.81 ± 0.16 vs. 30.09 ± 0.13 mm^3^, *t*_1,21_ = 1.35, *P* = 0.1910). **c** Genotype-dependent difference in relative amygdala volume does not exceed FDR (8.331 ± 0.145 vs. 8.693 ± 0.082 percent, *t*_1,21_ = 2.109, FDR *P* = 0.21). **d** No genotype-dependent difference in relative total hippocampus volume (4.842 ± 0.042 vs. 4.885 ± 0.0288 percent, *t*_1,21_ = 0.8351, *P* *=* 0.4131). **e** Coronal series highlighting regional relative size differences with significance exceeding FDR threshold = 0.05 (blue = smaller, red = larger). Statistics: *n* = 12 (wt) or *n* = 11 (het) adult brains as independent replicates, with bars representing mean ± SEM. Brain region size normalized to total brain volume. *t*-test for comparison of group means of 178 brain regions, with statistical significance assigned at F ≥ 4.0, corresponding to FDR *P* ≤ 0.05

Region-specific differences in brain volume and connectivity are frequently observed in human ASD patients and animal models by magnetic resonance imaging (MRI)^26,27^. Given that *Setd5*^+/−^ animals recapitulated select behaviors compatible with the human phenotype, anatomical imaging was undertaken to ascertain neuroanatomical-neurobehavioral correlations. Absolute and relative differences in brain volume were calculated by analysis of MRI from adult mouse brains. No difference beyond the FDR *P* < 0.05 was observed in total brain volume (*P* = 0.2606, Fig. 5a), total relative cortical volume (*P* = 0.1910, Fig. 5b), relative amygdala volume (*P* = 0.0471, FDR *P* = 0.21, Fig. 5c), or total relative hippocampus volume (*P* = 0.4131, Fig. 5d). However, when individual subregions were normalized to total brain volume, significant genotype-dependent differences were observed in several specific regions (Fig. 5e). Brain regions whose relative size differences exceeded the false discovery rate (FDR) *P* *=* 0.05 are overlaid in the coronal series (blue = smaller, red = larger, Fig. 5e). For absolute size differences not normalized to total brain volume, see Fig. S9. Specifically, the following autism-relevant brain regions emerged as regions of significant difference in *Setd5*^+/−^ brains beyond the FDR: secondary auditory cortex^28^ (larger, FDR *P* = 0.03), dorsolateral orbital cortex (larger, FDR *P* = 0.01) and frontal association cortex^29^ (larger, FDR *P* = 0.03). In the hippocampus, differences in the CA2 cell layer fell short of statistical significance (FDR *P* = 0.06). A comprehensive list of all subregions is displayed in Supplemental Sheet 3.

### Postnatal brain deficit in CTIP2^+^ cortical layer

We next performed a more detailed anatomical investigation. Evidence of aberrant cortical lamination has been observed in mouse models^17,18^ and proposed in human ASD^7^. To determine whether *Setd5*^+/−^ behavioral abnormalities in adults could be attributed to early lamination defects, we chose P1 and P10 brain sections stained for superficial cortical neuron marker Brn2 (purple) and deep cortical neuron markers Ctip2 (green) and Tbr1 (red)^30,31^ as described previously^17^ (Fig. S10A, B) for the ability to track development of cellular subpopulation composition. Cell layers were measured in images, at least 2 per animal, of coronal sections corresponding to the Allen Developing Mouse Brain Reference Atlas sections 90–140^32^ containing M1, at 30 and 70% from the dorsal midline. In P1 brain sections, no differences in thickness of the deepest or most superficial layers were observed: (TBR1^+^ layer, *P* = 0.9090; BRN2^+^ layer, *P* *=* 0.5906, Fig. S10C, G), although the CTIP2^+^ layer was significantly thinner in *Setd5*^+/−^ sections (144.2 ± 7.78 vs. 113.9 ± 6.25 μm, *P* *=* 0.0055, Fig. S10E). Similarly, in P10 brain sections, no differences in thickness of the deepest or most superficial layers were observed (TBR1^+^ layer, *P* = 0.0945, Fig. 6D; BRN2^+^ layer, *P* *=* 0.0883, Fig. S10G), although the CTIP2^+^ layer was again significantly thinner in *Setd5*^+/−^ sections (214.3 ± 7.11 vs. 178.4 ± 6.99 μm, *P* *=* 0.0012, Fig. S10E). Despite the differences in total layer thickness, no genotype-dependent differences were detected in cell density within the respective cortical layers at P1 or P10 (TBR1^+^ layer, *P* = 0.7481, *P* *=* 0.2191 Fig. S10D; CTIP2^+^ layer, *P* = 0.1064, *P* *=* 0.4667, Fig. S10F; BRN2^+^ layer, *P* *=* 0.5256, *P* *=* 0.1166 Fig. S10H).

## Discussion

*SETD5* has emerged as an intriguing ASD-risk candidate, guiding us to investigate the *Setd5* animal model to shed light on the contributions of this gene to neurodevelopment. Construct validity of the *Setd5* mouse with human ASD patients harboring *SETD5* mutations is of critical significance in extrapolating the findings of the animal model to human ASD etiology.

Findings from our in vitro and in vivo experiments echo published reports of existing animal models of autism and extend those findings at the level of physiology, which in the *Setd5*^+/−^ neurons and brain appear to converge on a picture of cortical hypoconnectivity. Synaptogenesis is impacted in various animal models of autism, from significant decreases in excitatory post-synaptic currents (mESPCs) among CA1 hippocampal neurons in neurexin-1α mice^33^ to increased synaptic density among cortical neurons in Fragile-X mice^34^. Cortical neurons from *Setd5*^+/−^ animals demonstrated reduced excitatory synaptic density. Importantly, this was not attributed to culture heterogeneity, as the CTIP2^+^ proportion of cells was comparable across genotypes in the current study. We observed significantly reduced neuritic outgrowth and soma size in cultured *Setd5*^+/−^ deep-layer cortical projection neurons. These deficits affected cortical layer V neurons positive for CTIP2, a population of cortical subprojection neurons implicated in human ASD pathogenesis^7^, which were found as a point of convergence among distinct ASD-related mutations^35^.

These data were corroborated by the reduced synchrony of network firing in *Setd5*^*+/−*^ cortical neurons. Significant genotype-dependent differences in high-frequency activity were observed over time, with focal differences in both low- and high-frequency activity specifically in the second week in vitro. These findings suggest a critical period of in vitro maturation during the second week in culture, in which control cultures showed both higher and more synchronized network activity, potentially leading to a more mature circuit via activity-dependent synapse formation and a sustained developmental difference persisting into the future.

Our transcriptomic analysis identified a subtle effect of *Setd5* haploinsufficiency on a specific cellular subpopulation in the developing brain, as assessed by differential gene expression in *Setd5*^+/−^ cortical neuronal cells. At the level of bulk RNA-Seq, downregulation of genes related to extracellular matrix organization was observed. A number of genes involving collagen synthesis, which is known to be dysregulated in models of ASD^36^, were also downregulated. Interestingly, the majority of these neurodevelopmental downregulated genes affected by *Setd5*^+/−^ occurred in cluster 3, a population of cells characterized by expression of inhibitory interneuron markers *Dlx1/Dlx2*^37^ and *Cdca7*, a gene that regulates intermediate progenitor production in the developing brain^38^. Significant downregulation of *Nnat* expression, involved in neuronal intracellular calcium signaling^39^, could connect to our finding of reduced calcium transient synchrony in *Setd5*^+/−^ cells. *Cdkn1c*, encoding cell cycle regulator p57, mediates cortical neurogenesis^40^ and was also significantly downregulated in the *Setd5*^+/−^ cells. *Malat1*, a long non-coding RNA (lncRNA) that was significantly downregulated in the *Setd5*^+/−^ cells, contributes to neurite outgrowth through the MAPK pathway during neuronal differentiation^41^ and could connect to our findings of neurite outgrowth restriction in *Setd5*^+/−^ neurons. Further analysis of *Setd5* deletion in specific cell types will elucidate the significance of *Setd5*-mediated transcriptomic abnormalities in the developing brain.

Extensive anatomical imaging data are available for both human ASD patients and genetic animal models. The most recent mega-analysis of human ASD MRI data found consistently decreased volume of the putamen, amygdala, and nucleus accumbens; and increased frontal but decreased temporal cortex thickness^42^. *Setd5*^+/−^ brains showed no evidence of overall macrocephaly, although significant volume differences were observed in several ASD-implicated brain subregions including regions of frontal cortex important for learning. An MRI analysis of 26 mouse models found consistent abnormalities of parieto-temporal and frontal lobes, cerebellar cortex, hypothalamus, and striatum, clustering the known models into three groups based on correlated size changes in distinct brain regions^26^. *Setd5*^+/−^ brains lacked the hallmarks of Ellegood et al.’s Group 1 (increased large white matter tract size) and Group 3 (decreased frontal cortex size) and thus may cluster approximately in Group 2 (decreased hippocampal size)^26^. These data provide an interesting insight into the downstream neuroanatomical consequences of *Setd5* haploinsufficiency, though it is premature to claim a causal relationship with observed behavioral phenotypes as the studies were not conducted in identical animals (i.e., correlating an individual animal’s behavior results with postmortem MRI analysis)^17^.

The *Setd5*^+/−^ adult animal demonstrated features compatible with human ASD, including those manifest by *SETD5* LoF human patients^3,4^. The three-chambered test of social interaction captures differences in social interaction preferences in numerous animal models of ASD^24^. Although *Setd5* het animals had normal social approach tendency, when faced with the task of discriminating between a familiar versus unfamiliar conspecific, mutant animals showed no preference for novel versus familiar, in contrast to controls. This pattern differs from other animal ASD models in that reduced sociability and lack of preference for social novelty were frequently observed together, including in the *Nlgn4, Slc6a4*, and *Gabrb3* models^24^, thus pointing to a more specific deficit in social recognition, rather than globally impaired socialization in the *Setd5*^+/−^ animals. The Barnes maze task detected a mild cognitive and spatial learning deficit in *Setd5*^+/−^ animals, a behavioral domain implicated in other ASD models^43^. Finally, the open field test revealed hyperactivity consistent with other mouse ASD models^44^, although the reduced thigmotaxis evidenced by increased time in the central area of the chamber, differed from other ASD models^45,46^.

In conclusion, we report here a new animal model for ASD, with autism-compatible CNS phenotypes at the cellular, network, and behavioral levels. Importantly, the animal model has validity with observed human ASD patients harboring *SETD5* mutations. The present model suggests that *Setd5* haploinsufficiency has a negative impact on early brain development, potentially through aberrant gene expression in select neuronal subpopulation(s) that contributes downstream to functionally hypoconnected cortical networks and behavioral abnormalities. Our data yield novel insights into the pathogenic basis for *SETD5* LoF ASD patients and provide a model for investigating the contribution of ASD susceptibility genes.

## Supplementary information

Figure S1

Supplemental Sheet 1

Supplemental Sheet 2

Supplemental Sheet 3

## Data Availability

The datasets generated during and/or analyzed during the current study are available from the corresponding author on reasonable request.

## References

[1] Pinheiro I (2012). Prdm3 and Prdm16 are H3K9me1 methyltransferases required for mammalian heterochromatin integrity. Cell.

[2] Osipovich AB, Gangula R, Vianna PG, Magnuson MA (2016). Setd5 is essential for mammalian development and the co-transcriptional regulation of histone acetylation. Development.

[3] Fernandes, I. R. et al. Genetic variations on SETD5 underlying autistic conditions. *Dev. Neurobiol*. **78**, 500–518 (2018). 10.1002/dneu.22584 29484850

[4] Grozeva D (2014). De novo loss-of-function mutations in SETD5, encoding a methyltransferase in a 3p25 microdeletion syndrome critical region, cause intellectual disability. Am. J. Hum. Genet..

[5] Kuechler A (2015). Loss-of-function variants of SETD5 cause intellectual disability and the core phenotype of microdeletion 3p25.3 syndrome. Eur. J. Hum. Genet..

[6] Pinto D (2014). Convergence of genes and cellular pathways dysregulated in autism spectrum disorders. Am. J. Hum. Genet..

[7] Stoner R (2014). Patches of disorganization in the neocortex of children with autism. N. Engl. J. Med..

[8] Zhang Y (2014). An RNA-sequencing transcriptome and splicing database of glia, neurons, and vascular cells of the cerebral cortex. J. Neurosci..

[9] Fukuda T, Itoh M, Ichikawa T, Washiyama K, Goto Y (2005). Delayed maturation of neuronal architecture and synaptogenesis in cerebral cortex of Mecp2-deficient mice. J. Neuropathol. Exp. Neurol..

[10] Chailangkarn T (2016). A human neurodevelopmental model for Williams syndrome. Nature.

[11] Beaudoin GM (2012). Culturing pyramidal neurons from the early postnatal mouse hippocampus and cortex. Nat. Protoc..

[12] Tabuchi K (2007). A neuroligin-3 mutation implicated in autism increases inhibitory synaptic transmission in mice. Science.

[13] Guyenet, S. J. et al. A simple composite phenotype scoring system for evaluating mouse models of cerebellar ataxia. *J. Vis. Exp*. (2010). http://www.jove.com/index/Details.stp?ID=1787, 10.3791/1787. 10.3791/1787 PMC3121238 20495529

[14] Katayama Y (2016). CHD8 haploinsufficiency results in autistic-like phenotypes in mice. Nature.

[15] Crawley JN (2007). Mouse behavioral assays relevant to the symptoms of autism. Brain. Pathol..

[16] Yang, M., Silverman, J. L. & Crawley, J. N. Automated three-chambered social approach task for mice. *Curr. Protoc. Neurosci.* **Chapter 8:** Unit 8 26 (2011). 10.1002/0471142301.ns0826s56 PMC4904775 21732314

[17] Gompers AL (2017). Germline Chd8 haploinsufficiency alters brain development in mouse. Nat. Neurosci..

[18] de la Torre-Ubieta L, Won H, Stein JL, Geschwind DH (2016). Advancing the understanding of autism disease mechanisms through genetics. Nat. Med..

[19] McCabe AK, Chisholm SL, Picken-Bahrey HL, Moody WJ (2006). The self-regulating nature of spontaneous synchronized activity in developing mouse cortical neurones. J. Physiol..

[20] Manning JR, Jacobs J, Fried I, Kahana MJ (2009). Broadband shifts in local field potential power spectra are correlated with single-neuron spiking in humans. J. Neurosci..

[21] Gleichmann M, Mattson MP (2011). Neuronal calcium homeostasis and dysregulation. Antioxid. Redox Signal..

[22] Schmunk G, Gargus JJ (2013). Channelopathy pathogenesis in autism spectrum disorders. Front. Genet..

[23] Satija R, Farrell JA, Gennert D, Schier AF, Regev A (2015). Spatial reconstruction of single-cell gene expression data. Nat. Biotechnol..

[24] Silverman JL, Yang M, Lord C, Crawley JN (2010). Behavioural phenotyping assays for mouse models of autism. Nat. Rev. Neurosci..

[25] McKinstry SU (2014). Huntingtin is required for normal excitatory synapse development in cortical and striatal circuits. J. Neurosci..

[26] Ellegood J (2015). Clustering autism: using neuroanatomical differences in 26 mouse models to gain insight into the heterogeneity. Mol. Psychiatry.

[27] Yang DY, Beam D, Pelphrey KA, Abdullahi S, Jou RJ (2016). Cortical morphological markers in children with autism: a structural magnetic resonance imaging study of thickness, area, volume, and gyrification. Mol. Autism.

[28] Edgar JC (2015). Auditory encoding abnormalities in children with autism spectrum disorder suggest delayed development of auditory cortex. Mol. Autism.

[29] Sawa T (2013). Dysfunction of orbitofrontal and dorsolateral prefrontal cortices in children and adolescents with high-functioning pervasive developmental disorders. Ann. Gen. Psychiatry.

[30] Zhang J, Jiao J (2015). Molecular biomarkers for embryonic and adult neural stem cell and neurogenesis. Biomed. Res. Int..

[31] Aldiri I (2017). The dynamic epigenetic landscape of the retina during development, reprogramming, and tumorigenesis. Neuron.

[32] Science AIfB. *Allen Developing Brain Reference Atlas*, Allen Institute, Seattle, WA (2008). http://developingmouse.brain-map.org/static/atlas.

[33] Etherton MR, Blaiss CA, Powell CM, Sudhof TC (2009). Mouse neurexin-1alpha deletion causes correlated electrophysiological and behavioral changes consistent with cognitive impairments. Proc. Natl Acad. Sci. USA.

[34] Comery TA (1997). Abnormal dendritic spines in fragile X knockout mice: maturation and pruning deficits. Proc. Natl Acad. Sci. USA.

[35] Willsey AJ (2013). Coexpression networks implicate human midfetal deep cortical projection neurons in the pathogenesis of autism. Cell.

[36] Olde Loohuis NFM (2017). Altered expression of circadian rhythm and extracellular matrix genes in the medial prefrontal cortex of a valproic acid rat model of autism. Prog. Neuropsychopharmacol. Biol. Psychiatry.

[37] Xu Q, Cobos I, De La Cruz E, Rubenstein JL, Anderson SA (2004). Origins of cortical interneuron subtypes. J. Neurosci..

[38] Huang YT, Mason JO, Price DJ (2017). Lateral cortical Cdca7 expression levels are regulated by Pax6 and influence the production of intermediate progenitors. Bmc. Neurosci..

[39] Lin HH (2010). Neuronatin promotes neural lineage in ESCs via Ca(2+) signaling. Stem Cells.

[40] Pfurr S (2017). The E2A splice variant E47 regulates the differentiation of projection neurons viap57(KIP2) during cortical development. Development.

[41] Chen L (2016). Long non-coding RNA Malat1 promotes neurite outgrowth through activation of ERK/MAPK signalling pathway in N2a cells. J. Cell. Mol. Med..

[42] van Rooij, D. et al. Cortical and subcortical brain morphometry differences between patients with autism spectrum disorder and healthy individuals across the lifespan: results from the ENIGMA ASD working group. *Am. J. Psychiatry* **175**, 359–369 (2017). 10.1176/appi.ajp.2017.17010100 PMC6546164 29145754

[43] Blundell J (2010). Neuroligin-1 deletion results in impaired spatial memory and increased repetitive behavior. J. Neurosci..

[44] Peier AM (2000). Over)correction of FMR1 deficiency with YAC transgenics: behavioral and physical features. Hum. Mol. Genet..

[45] Dachtler J (2014). Deletion of alpha-neurexin II results in autism-related behaviors in mice. Transl. Psychiatry.

[46] Hiramoto T (2011). Tbx1: identification of a 22q11.2 gene as a risk factor for autism spectrum disorder in a mouse model. Hum. Mol. Genet..

