# Supplementary material for: Setd5 haploinsufficiency alters neuronal network connectivity and leads to autistic-like behaviors in mice
Source: Transl Psychiatry. 2019 Jan 17;9:24. doi: 10.1038/s41398-018-0344-y (PMC6336863; doi:10.1038/s41398-018-0344-y)

SUPPLEMENTAL METHODS

Primary cortical neuron culture

Primary cortical neurons from embryonic day 18.5 (E18.5, from timed matings) or postnatal day 0 (P0) offspring of *Setd5*$^{+/+}$ x *Setd5*$^{+/-}$ were harvested as described previously[1]. Cells were plated on poly-ornithine (PO, 10μg/mL) and laminin (1μg/mL) coated tissue-culture plates. Culture density was determined by application (immunocytochemistry/Ca^{2+} imaging: 50k cells/well of 96well (w) plate; multielectrode array: 200k cells/64 electrode array). Cells were cultured in Neurobasal medium + B27 supplement (Gibco) and cultured *in vitro* up to 5 weeks. To arrest glial proliferation in preparations for immunocytochemistry, cytosine arabinoside (araC, Tocris #4520, Tocris, Bristol, UK) was added to all cultures at 2 μM for 24 hours beginning at 3 DIV.

Immunohistochemistry/Immunocytochemistry

Tissue for histopathological or immunohistochemical analysis was processed as follows. Animals were deeply anesthetized (ketamine 100mg/kg and xylazine 10mg/kg, intraperitoneal [IP]), and perfused transcardially with 4% paraformaldehyde (PFA) in phosphate-buffered saline (PBS), with brains cryoprotected overnight in 30% sucrose for histological and immunohistochemical analysis. Brain tissue was frozen and sectioned into 5μm (histology) or 30μm (immunohistochemistry) thick sections. Immunohistochemistry was performed on coronal sections through M1 cortex, in comparable anatomical regions across groups, with permeabilization (15 minutes, room temperature, 0.25% Triton-X in PBS followed by 3x washes in PBS), blocking (4 hours, 4°C, 3% bovine serum albumin (BSA) in PBS), primary antibody (Supplemental Table 1

for dilutions in 1% BSA in PBS, overnight, 4°C, followed by 3x PBS washes), secondary antibody, and nuclear staining (4',6-Diamidino-2-Phenylindole (DAPI), 0.1μg/mL in PBS, 10 minutes, room temperature). Cortical layer thickness measurements were performed as described previously[2], as the average between measurements perpendicular to the cortical surface taken at 30% and 70% from the dorsal midline. For cells stained by immunocytochemistry, for morphological analysis neurons were analyzed by Neurolucida Explorer (MBF Bioscience, Williston, VT, USA) for the metrics of soma size, total neurite length and number, and Sholl analysis. Synaptic puncta were counted as colocalized overlapping HOMER1^{+} and VGLUT1^{+} puncta along randomly selected MAP2^{+} neurites as described previously[3]. To ensure standardization of analysis, only cells with a pyramidal soma shape, with at least 2 neurites emanating from the cell body were included. As a quantification of cell death, cells were also processed by terminal deoxynucleotidul transferase dUTP nick end labeling (TUNEL, Click-iT TUNEL Imaging Assay, Life Technologies, Carlsbad, CA, USA). Briefly, fixed permeabilized cells were exposed to the terminal deoxynucleotidyl transferase enzyme, which can incorporate a modified dUTP nucleotide into damaged cellular DNA. Cells were then exposed to the click copper-catalyzed detector and imaged by fluorescence microscopy, with labeled cells considered to be apoptotic. Statistical comparisons were conducted by unpaired, 2-tailed *t*-test. To avoid confounding from the GFP transgene in *Setd5*$^{+/-}$ tissues or primary cells, no-secondary antibody negative controls were employed in the 488 nm channel in all experiments. While GFP could be detected by staining with an anti-GFP primary antibody, no endogenous GFP expression was observed. Stained sections

were mounted (Fluoromount) for fluorescence microscopy (Zeiss Apotome Microscope, Oberkochen, Germany). Sample size was selected from an initial pilot study of neurons from *n*=4 animals per genotype.

Electrophysiology

MEA: Cells analyzed by MEA electrophysiology were plated at 250k cells/MEA of 12w plate (Axion Biosystems, Atlanta, GA, USA) and cultured up to 32 DIV. Beginning at 8 DIV, recordings were conducted every 1-3 days through 32 DIV on the Axion Maestro system (Axion). Recordings measured spontaneous electrical spiking activity, 5.5 standard deviations (σ) above noise through a 0.1-5000 Hz filter, proceeded 10min, and discarded the initial 60 seconds (s) from the analysis. Raw data were analyzed by the NeuralMetrics tool (Axion). Weighted mean firing represented the mean firing rates from only active electrodes (5 spikes/min); normalized burst frequency defined as 5 spikes with maximum inter-spike interval (ISI) of 100 μs; electrodes per burst defined as the number of electrodes participating in a single bursting event; and synchrony index as a unitless measure of firing synchrony, between 0-1, with 1=greatest synchrony, as a metric of inter-electrode cross correlogram of firing events (Supplemental Table 2). For local field potential (LFP) computation, raw 64-channel MEA recordings (12500 Hz) were low-pass filtered (300 Hz) and down-sampled to 1000 Hz (MATLAB *resample.m*). Power spectral density (PSD) were computed using Welch's method (*pwelch.m*) with window length of 2-seconds and step length of 1-second. For each culture, a single recording yields a 64-by-1000 power matrix, where 64 represents the number of MEA channels and 1000 represents the number of frequencies (0-500Hz at 0.5Hz resolution).

For power comparison over time, baseline spectral power was taken to be the first day of recording (3 DIV), and all subsequent PSDs were normalized per frequency to 3 DIV and logged to compute log_{10} power ratio. First recording, by definition, has log power ratio of 0 at every frequency. Band-specific log_{10} power ratio were computed as the following: first take the normalized median log_{10} power ratio over the specified frequencies (1-10 Hz for low frequency, 100-150 Hz for high frequency), then compute the median over all 64 channels. Finally, compute the mean and standard deviation over samples from the same condition. 2-way ANOVA (*anova2.m*) was run on the above genotype-pooled power ratios over time. At the end of the experiment, since typical action potentials are triggered by voltage-gated sodium channels, tetrodotoxin (TTX, 10nM) was added to the cultures and spikes measured. Data were representative of several experiments combined, with neurons from at least *n*=12-13 animals per genotype. Statistics were analyzed as 2-way ANOVA for the factors genotype and DIV. MATLAB code proprietary.

Fluorescent Ca^{2+} Imaging: Imaging experiments were performed on a Zeiss inverted microscope equipped with a fluorescein filter set (excitation 485/20 nm, emission 525/30 nm). Images were acquired at 30-msec intervals for 20-30 seconds using a CCD camera (Orca Flash, Hamamatsu, Japan). To detect dynamic changes in intracellular calcium concentration, we used Fluo-4 AM, a fluorescent calcium indicator (Life Technologies). Cultured cortical neurons (14-21 DIV) were loaded with 2 μM Fluo-4AM for 30 minutes in the CO_2 incubator in loading buffer (132 mM NaCl; 4.2 mM KCl; 1.8 mM CaCl2, 5 mM D-glucose, 10 mM HEPES; adjusted to pH 7.4) or in the conventional

cell culture media. Subsequently, cells were left at room temperature for additional 15 minutes to achieve a complete de-esterification of the dye. After the incubation with the dye, neurons were washed 3 times with the conventional media and were stored in the CO_2 incubator for additional 10 min to recover. During experiments, we recorded the spontaneous network activity of wt or het neurons for the same period of time, using a live-cell imaging chamber with CO_2 and 37°C incubation. Acquisition and analysis were performed using Zen Pro software (Zeiss) and FIJI 2 software. To extract the data from raw movies, a user manually determined regions of interest (ROIs) that exhibited considerable activity-driven intensity changes, and plotted changes in intensity vs. time. Fluorescence intensity was measured over the entire cell body, and responses were presented as arbitrary units (AU). For neuronal activity correlation analysis, we have developed the custom-made algorithm (available upon request) using OriginPro 2017 platform (OriginLab, Northhampton, MA, USA). Briefly, we use the rolling window of a predetermined length to analyze the signal intensity trace from each individual neuron. The algorithm calculates the ratio of the maximal value of the fluorescent signal of the (N+1) test window in the image sequence to the average value of the test window (N). By comparing the several calculated values in succession, this approach accurately determines the initial moment of the fluorescent signal intensity increase. By calculating the first derivative of the intensity signal, we have confirmed the accuracy of this approach. Next, we are identifying the correlation between all the individual fluorescent signal traces. Two or more traces are considered to be correlated, when the fluorescent signal action potential initialization will be (t±1) where "t" is an individual time point. In

order to compare the level of correlated neuronal activity for individual wells with the different number of identified neurons in each well, we are using the normalized correlated value for our test samples. Sample size was selected from an initial pilot study of n=3 wells per genotype.

Electroencephalogram (EEG) Recordings: All animal protocols were approved by the Cleveland Clinic Institutional Animal Care and Use Committee. All experiments were conducted in accordance with the principles and procedures outlined in the National Institutes of Health Guidelines for the Care and Use of Experimental Animals. *Setd5*$^{+/-}$ and *Setd5*$^{+/+}$ littermates were used. Animals were anesthetized with a mixture of ketamine (100 mg/kg, ACE Surgical Supply Co, Brockton, MA, USA) and xylazine (10 mg/kg, Sigma-Aldrich, St. Louis, MO, USA) and placed in a stereotactic apparatus (David Kopf Instruments, Tujunga, CA, USA). For hippocampal recordings, pedestal gold-plated stainless-steel electrodes (0.2 mm in diameter; PlasticsOne, New York, NY, USA) were placed bilaterally in the intermediate regions of hippocampi (LH, left hippocampus; RH, right hippocampus; 2.48 mm posterior to the bregma, 2.63 mm lateral from the midline, and 3.38 mm deep from the dura). Stainless-steel screws (E363/96/2.4/SPC; PlasticsOne, New York, NY, USA) were placed on the dura mater over bilaterally frontal motor cortex (LC, left primary motor-cortex; RC, right primary motor-cortex, 0.5 mm posterior to the bregma, 2.45 mm lateral from the midline). An additional stainless-steel screw was placed just to the right of the frontal sinus and served as a referential electrode. The five electrodes were then connected to a plastic plug (MS363; 6 Channel, PlasticsOne, New York, NY, USA), which was fixed to the

skull with dental cement (Orthodontic Resin liquid (651002) and powder (651006); Caulk®, Milford, DE, USA). Mice were left unrestrained for 5 days to allow for recovery from surgery before further manipulation, and prolonged EEG recording were performed. Continuous EEG recording and video monitoring was performed on each mouse. During EEG monitoring, mice had free access to food and water ad libitum. EEG (CEEG) was monitored for 16 hours/day for 3 consecutive days. EEG data were acquired and analyzed by using a Vangard system (Vangard, Cleveland, OH, USA). EEG sampling rate was 100 Hz, and the recording filter was set at 5.3 Hz and 30 HZ for low- and high-frequency, respectively. The correlations between EEG events and overt behavioral responses were assessed by using split screen video monitoring system. Epileptic spikes and seizure activities were analyzed from EEG data. Epileptic spikes (ictal spikes) were defined as paroxysmal electrical activity: they last 20-150 ms and their amplitude is greater than two times of average background EEG activity. Seizure activity is scored when epileptic spikes persist longer than 10 seconds. Seizure frequency and duration was measured. Once EEG recording was complete, mice were sacrificed and the location of electrodes was confirmed with H&E staining. Sample size was chosen as an initial pilot experiment to detect any genotype-dependent effects.

RNA-Seq/scRNA-Seq

Sorting: Primary cortical neurons from E18.5 fetuses were harvested into suspension using 1 μM flavopiridol in the dissection and digestion buffers, then stained with anti-CD24 and anti-CD45 antibody cocktail. Cells were sorted on a Sony LE-SH800SZFCPL

and analyzed by FlowJo 10.4. Neurons/progenitors were defined as: FSC high, SSC low, $CD24^+$ $CD45^-$, singlets (FSC-H vs. FSC-W), $DAPI^-$.

RNA-Seq: RNA-Sequencing Library Preparation Sequencing libraries were prepared from polyA enriched mRNA, either as previously described or as follows. Poly A enriched mRNA was fragmented, in 2x Superscript III first-strand buffer with 10mM DTT (Invitrogen), by incubation at 94°C for 9 minutes, then immediately chilled on ice before the next step. The 10 μL of fragmented mRNA, 0.5 μL of Random primer (Invitrogen), 0.5 μL of Oligo dT primer (Invitrogen), 0.5 μL of SUPERase-In (Ambion), 1 μL of dNTPs (10 mM) and 1 μL of DTT (10 mM) were heated at 50°C for three minutes. At the end of incubation, 5.8 μL of water, 1 μL of DTT (100 mM), 0.1 μL Actinomycin D (2 μg/μL), 0.2 μL of 1% Tween-20 (Sigma) and 0.2 μL of Superscript III (Invitrogen) were added and incubated in a PCR machine using the following conditions: 25°C for 10 minutes, 50°C for 50 minutes, and a 4°C hold. The product was then purified with RNAClean XP beads according to manufacturer's instruction and eluted with 10 μL nuclease-free water. The RNA/cDNA double-stranded hybrid was then added to 1.5 μL of Blue Buffer (Enzymatics), 1.1 μL of dUTP mix (10 mM dATP, dCTP, dGTP and 20 mM dUTP), 0.2 μL of RNAse H (5 U/μL), 1.05 μL of water, 1 μL of DNA polymerase I (Enzymatics) and 0.15 μL of 1% Tween-20. The mixture was incubated at 16°C for 1 hour. The resulting dUTP-marked dsDNA was purified using 28 μL of Sera-Mag Speedbeads (Thermo Fisher Scientific), diluted with 20% PEG8000, 2.5M NaCl to final of 13% PEG, eluted with 40 μL EB buffer (10 mM Tris-Cl, pH 8.5) and frozen -80°C. The purified dsDNA (40 μL) underwent end repair by blunting, A-tailing and adapter ligation as previously

described[4] using barcoded adapters (NextFlex, Bioo Scientific). Libraries were PCR-amplified for 9-14 cycles, size selected by gel extraction, quantified Qubit dsDNA HS Assay Kit (Thermo Fisher Scientific) and sequenced on either a Genome Analyzer II (Illumina) or Hi-Seq 2000 (Illumina) for 51 cycles.

All sequencing was conducted using an Illumina Hi-Seq 4000 sequencer using single-end 75bp reads. All data were aligned to the mm10 assembly of the mouse genome, and all subsequent data analysis was performed using HOMER, and detailed instructions for analysis can be found at http://homer.salk.edu/homer/. Each sequencing experiment was normalized to total of 10^7 uniquely mapped tags by adjusting the number of tags at each position in the genome to the correct fractional amount given the total tags mapped. Sequence experiments were visualized by preparing custom tracks for the UCSC genome browser. Differentially expressed genes were identified using HOMER as described previously[5]. Gene ontology was performed using the web based functional annotation tool Metascape[6].

sc-RNA-Seq: Library construction and sequencing: Primary cortical neurons from E18.5 fetuses were isolated as described above. Single cell suspensions were loaded onto the 10X Genomics Chromium Controller instrument to generate single cell GEMs, targeting 5000 cells per sample. GEM-RT and library construction were performed following the 10X Genomics Protocol (Single Cell 3' Reagent Kits V2). Library fragment size distributions was determined using an Agilent Bioanalyzer High Sensitivity chip, and library DNA concentrations were determined using a Qubit 2.0 Fluorometer (Invitrogen).

Libraries were sequenced to a depth of approximately 50,000 paired end reads per cell using an Illumina HiSeq4000.

Data Preprocessing and Quality Control: Data was mapped to the mouse mm10 genome and aggregated using Cell Ranger V2.1 (10X Genomics)[7]. Data was filtered, processed, and analyzed using the Seurat R toolkit version 2.3.0 for single cell genomics[8]. Initial pre-processing thresholds were set to include genes detected in at least 3 cells and cells with at least 200 genes. Additional pre-processing was performed after plotting distributions of number of genes (nGenes), number of unique molecular identifiers (nUMI), and percent mitochondrial transcripts (percent.mito) per cell. The following thresholds were then applied: nGene > 600 and < 5000, nUMI > 500 and < 15000, and percent.mito < 0.06. A total of 6562 cells passed these thresholds and were used for downstream analyses. Data was then normalized using the NormalizeData function with parameters normalization.method = "LogNormalize", scale.factor = 10000.

Clustering and Analysis: Variable genes were identified with the FindVariableGenes function with parameters mean.function = ExpMean, dispersion.function = LogVMR, x.low.cutoff = 0.0125, x.high.cutoff = 3, y.cutoff = 0.5, which identified 1548 variable genes. Data was then scaled using the ScaleData function, with the “vars.to.regress” argument including nUMI and percent.mito to remove these unwanted sources of variation. A total of 100 principal components were calculated from these variable genes using the function RunPCA. Selection of significant PCs to include in further analyses was determined using the JackStraw method with the num.replicate parameter set to 200, along with visualization of each PC using the PCHeatmap function. 61 total PCs

with p < 1e-5 were included. Clusters were then identified using the FindClusters function with arguments reduction.type = "pca", dims.use = sigPCs, resolution = 0.6, which identified 14 clusters of cells. Quality control of clusters and samples was performed by plotting nGene and nUMI, which confirmed no major differences based on these parameters. The TSNEPlot function was used to generate t-SNE[9] plots to visualize data by cluster and by sample of origin. Marker genes for each cluster was determined using the FindAllMarkers function with parameters only.pos = TRUE, min.pct = 0.25, and thresh.use = 0.25. Based on these marker genes, clusters 0, 1, 3, 4, 5, and 8 were selected for further analysis. A heatmap of the top 20 marker genes from these clusters was generated with function "DoHeatmap" with max.cells.per.ident set to 100. To identify genes with significantly different gene expression due to genotype within each cluster, the FindMarkers function was used arguments min.pct = 0.1, logfc.threshold = 0.25, test.use = "MAST". Data were converted from natural log to log2 fold change values prior to plotting, and X- and Y-linked transcripts were excluded from plots.

Animals

The *Setd5*^{GFP} transgenic mouse, hereafter referred to as the *Setd5*$^{+/-}$ heterozygous knockout animal, was obtained and crossed for 5 generations onto the C57/Bl6 background[10]. Animals were housed in same-sex, single-genotype cages, 5 mice per cage, on a light/12 hour dark cycle with access to chow and water *ad libitum*. Statistical analysis for behavioral experiments was conducted by *t*-test of pooled male-female group means, or 2-way ANOVA for experiments with several factors (eg, genotype and

trial), with wild-type littermates serving as controls. Behavioral experiments and offline analyses were carried out by a blinded observer, and other than tests requiring consecutive days, animals were permitted at least 4 days rest between different behavioral tasks. All breeding, husbandry, behavioral, and euthanasia manipulations were carried out in accordance with the Institutional Animal Care and Use Committee (IACUC) at the University of California, San Diego. Sample sizes were determined by an initial pilot experiment of n=6 animals per genotype.

Neurologic and Metabolic Assays

Weight: animals were weighed once at 10 weeks age.

Grip strength: 10-week-old animals were assayed for grasping strength on the grip strength grid inserted into the Omnitech Animal Grip Strength Meter (Columbus, OH, USA). The mean of three measurements was recorded and normalized by body weight.

Neurological score: 10 week old animals were assayed on a composite neurologic scoring scale as described previously[11]. Briefly, each animal was observed in 4 separate tasks (ledge test, hindlimb clasping, gait, kyphosis) and scored from 0 (unaffected) to 3 (severely affected), with a maximum total of 12 per mouse.

Rotorod: 10-week-old animals were subject to the rotorod test of motor learning and coordination as described previously[12]. The trials were conducted on an accelerating rotorod, (Columbus Instruments, Columbus, OH, USA), with 4 trials per day over 2 consecutive days, with constant acceleration from 0-40rpm over 60 seconds, with 100 seconds total per trial.

Behavioral Assays

Open field: The open field test was conducted as described previously[12]. The task itself is conducted in an open field apparatus (Omnitech Electronics, Columbus, OH, USA) plexiglass box, 48cm x 48cm containing photo beams that detect motion. The 11-week old naïve animal is placed in the box and allowed to move freely for the 1 hour test period. Automated tracking of locomotion in the 3 consecutive 20-minute periods is plotted as a metric of animal activity. For purposes of measuring thigmotaxis, or the animal's preference for the perimeter, the chamber was divided into the central 25% and peripheral 75% regions.

Barnes maze: Assays were conducted as described previously[13], at 16 weeks age. Specifically, the Barnes maze was a 1m diameter, flat white plastic disk. Around the perimeter were 20 symmetrical holes, one of which (the target) contained a closed plastic escape box filled with fresh animal cage bedding for each trial. The maze itself was elevated above the ground 1m. The position of the escape chamber relative to the room was randomized for each mouse but remained the same across trials per animal. In each 3-minute trial, the animal was placed in the center of the maze in the dark, the room was illuminated, and latency to solve the maze and enter the escape chamber was measured. Following an initial 1 minute acquisition trial to acquaint the mouse with the maze and escape chamber, 4 trials per mouse per day were conducted for 4 consecutive days. A probe trial was conducted on day 5 and day 12 in which the hole covering the escape chamber was closed, to confirm that the animal indeed used spatial cues from the room. The maze was cleaned and disinfected to remove any olfactory cues between all trials.

Elevated plus maze: The elevated plus maze was conducted as described previously[14]. The plus maze was constructed of opaque plastic and elevated 1m above the ground; it consists of 4 33x5cm arms, two of which are enclosed with 25cm high walls. During the single 10-minute test period, the 11 week old animal was placed in the center of the maze and allowed to move freely[12]. Time spent in the open and closed arms of the maze was measured offline by video analysis.

Nest building: In this single overnight (8 hour) test[13], the 12-week-old animal was singly housed in a clean cage with fresh bedding with a pressed cotton Nestlet (Ancare, Bellmore, NY, USA). In the morning, the nest formed was visually inspected and assigned a score (1=untouched; 2=partially torn; 3=mostly shredded, but no single nest site; 4=single but flat nest; 5=single, 3-dimensional, complex nest).

3-chamber social interaction: The 3-chamber test was conducted on 13-14-week-old animals as described previously[13, 15]. The box itself was 20x40x22cm, with clear plexiglass walls and 2 plexiglass interior dividers to separate the 3 separate chambers; a hole in the dividers permits free movement between them. In all tests, the subject animal was placed in the empty center chamber and allowed to move freely through the 10 min test period, with the time spent in each chamber (left or right) measured by offline video analysis, with the contents of each chamber (left vs. right) randomized for each experimental mouse. In the first test, for sociability and social approach, one chamber contained a sex- and age-matched but unfamiliar mouse (stranger 1) contained within a smaller wire mesh container permitting reciprocal snout-snout interactions; the other chamber contained a similar container, but empty. In the second

test, for social-novelty preference, the initial unfamiliar mouse remained contained in the wire mesh container and now served as an investigated unfamiliar mouse (stranger 1). The second, previously empty chamber was now filled with a new, age- and sex-matched but completely unfamiliar mouse (stranger 2) to the experimental mouse. In the third test, for social-preference, one chamber was filled with an unfamiliar sex- and age-matched mouse (stranger 3), while the other was filled with a cagemate animal.

GenotypingAnimal genotyping was conducted using the Kapa Hot-Start genotyping kit (Kapa Biosystems, Wilmington, MA, USA). Primers for *Setd5* are as follows: FWD Acgtttccgacttgagttgc and REV atactccgaggcggatcac.

MRI

For magnetic resonance imaging analysis, *n*=12 per group (*Setd5*$^{+/+}$ and *Setd5*$^{+/-}$) of 8 week female mice were deeply anesthetized (ketamine: 150mg/kg and xylazine 10mg/kg) and perfused in PBS with 2mM gadoteridol (ProHance, Bracco Diagnostics, Monroe Township, NJ, USA) followed by 4% PFA with 2mM gadoteridol. Skulls were dissected and brains imaged by MRI. A multi-channel 7.0 Tesla MRI scanner (Varian Inc., Palo Alto, CA, USA) was used to image the brains within their skulls. Sixteen custom-built solenoid coils were used to image the brains in parallel[16]. In order to detect volumetric changes, we used the following parameters for the MRI scan: T2-weighted, 3-D fast spin-echo sequence, with a cylindrical acquisition of k-space, a TR of 350 ms, and TEs of 12 ms per echo for 6 echoes, field-of-view equaled to 20 x 20 x 25 mm3 and matrix size equaled to 504 x 504 x 630. Our parameters output an image with 0.040 mm isotropic voxels. The total imaging time was 14 hours. To visualize and

compare any changes in the mouse brains the images are linearly (6 followed by 12 parameter) and non-linearly registered together. Registrations were performed with a combination of mni_autoreg tools[17] and ANTS (advanced normalization tools)[18, 19]. All scans are then resampled with the appropriate transform and averaged to create a population atlas representing the average anatomy of the study sample. The result of the registration is to have all images deformed into alignment with each other in an unbiased fashion. For the volume measurements, this allows for the analysis of the deformations needed to take each individual mouse's anatomy into this final atlas space, the goal being to model how the deformation fields relate to genotype[20, 21]. The jacobian determinants of the deformation fields are then calculated as measures of volume at each voxel. Significant volume differences can then be calculated by warping a pre-existing classified MRI atlas onto the population atlas, which allows for the volume of 182 different segmented structures encompassing cortical lobes, large white matter structures (i.e. corpus callosum), ventricles, cerebellum, brain stem, and olfactory bulbs[20, 22-24] to be assessed in all brains. Further, these measurements can be examined on a voxel-wise basis in order to localize the differences found within regions or across the brain. Multiple comparisons in this study were controlled for using the False Discovery Rate[25].

Microscopy

Fluorescence microscopic images were acquired using Zen software on an inverted microscope (Zeiss Apotome) with 3-dimensional reconstruction of 10-20 μm composite z-stack images. The objective ranged from 10x (brain tissue immunohistochemistry) to

40x/63x (neuronal morphology and synaptic puncta). Illumination was provided by fluorescent bulb with appropriate filters for DAPI, AlexaFluor 488, AlexaFluor 555, or AlexaFluor 647 fluorophores. Zen Apotome deconvolution was applied to all raw images prior to analysis.

Statistics

Detailed statistical methods are included in the description of individual experiments. Statistical analysis was conducted with GraphPad Prism 7 software. The α threshold for statistical significance was set at 0.05. In general, for comparison of 2 group means, 2-tailed, unpaired *t*-test was used (Mann-Whitney test for ordinal data). For comparison of 2 more or more factors (eg, genotype and sex), 2-way ANOVA was used. For MRI data, a FDR *P* value of 0.05 was used to evaluate significance of *P* values obtained by *t*-test. Randomization was not performed prior to experiments, and group assignment was based on genotype. In all cases, the experimenter was blinded during data acquisition and analysis. Significance level was defined as $^{*}P<0.05$, $^{**}P<0.01$, $^{***}P<0.001$, $^{****}P<10^{-4}$.

Data availability

The datasets generated during and/or analyzed during the current study are available from the corresponding author on reasonable request.

SUPPLEMENTAL FIGURE LEGENDS

Supplemental Figure 1 Cell culture quality control. **A)** No difference in percent apoptotic neurons (P=0.3220, $t_{1,45}$=1.001, DF=45). **B)** No differences in percent excitatory (VGLUT1^{+}, P=0.2821, $t_{1,16}$=1.113, DF=16) or inhibitory neurons (GAD65/67^{+}, P=0.2700, $t_{1,16}$=1.143, DF=16). **C)** No differences in percent neurons (NeuN^{+} MAP2^{+}, P=0.7181, $t_{1,30}$=0.3644, DF=30) or glia (GFAP^{+}, P=0.7181, $t_{1,30}$=0.3644, DF=30). **D)** No differences detected in percent CTIP2^{+} neurons (48.43±3.40 vs. 51.60±4.11 percent, P=0.5571, $t_{1,28}$=0.5943, DF=28). **E-F)** Images of primary neuron cultures plated atop MEA, scale bar=400 µm. A=31 *Setd5*$^{+/+}$ and 16 *Setd5*$^{+/-}$ images, with group means compared by *t*-test. B=9 each *Setd5*$^{+/+}$ and *Setd5*$^{+/-}$ images, with groups means compared by *t*-test. C=19 *Setd5*$^{+/+}$ and 13 *Setd5*$^{+/-}$ images, with groups means compared by *t*-test. D=15 each *Setd5*$^{+/+}$ 15 *Setd5*$^{+/-}$ images, with groups means compared by *t*-test. Replicates as individual images with error bars representing mean±SEM.

Supplemental Figure 2 Depiction of Ca^{2+} labeling by Fluo-4AM *in vitro.* **A-B)** Representative images depicting circled regions of interest (ROIs) in wt (A) and het (B) primary neuron cultures for quantification of calcium signaling, scale bar 50 µm. **C)** Trace of spontaneous Ca^{2+} signal depicting synchronized firing activity in neurons.

Supplemental Figure 3 FACS sorting strategy for E18.5 cortical progenitors. **A)** Cells defined as high FSC and low SSC **B)** Cortical neurons defined as $CD24^{+}$ and $CD45^{-}$ **C)** Singlets and **D)** Live ($DAPI^{-}$) cells.

Supplemental Figure 4 Gene ontology analysis of significantly downregulated genes from bulk RNA-Seq of sorted E18.5 $CD24^{+}$ $CD45^{-}$ cortical progenitors. Statistics: cells from *n*=5 (*Setd5*$^{+/+}$) and *n*=5 (*Setd5*$^{+/-}$) mice sorted cortical suspensions.

Supplemental Figure 5 Single-cell RNA-Seq clustering of sorted cortical progenitors. **A)** Clustering of pooled wt and het reads into clusters of interest (0,1,3,4,5,8) by expression of top 20 genes (right). **B)** Split dot-plot display of differential expression of neuronal progenitor subpopulation markers by t-SNE cluster and genotype. Dot size represents proportion of expressing cells; color represents relative expression.

Supplemental Figure 6. Behavioral data separated by sex and genotype. **A-C)** Three-chamber social interaction task, measured by ratio of time spent per chamber for (A) sociability, (B) social-novelty preference, or (C) social preference. **D)** Total locomotion distance traveled in open field task. **E)** Time spent in central 25% of open field chamber. **F)** Percent time spent in open arms of elevated plus maze. **G)** Nest complexity scores in nest building task. **H)** Latency to solve maze in Barnes maze task. Statistics: expanded statistics found in Table S3. *n*=10 total animals per sex, per genotype; 2-way ANOVAs for factors sex, genotype, and trial or time where applicable. Graphs are separated male

and female data for each genotype, with replicates as individual animals and error bars representing mean±SEM.

Supplemental Figure 7 No metabolic or neurologic deficits observed in *Setd5*$^{+/-}$ adult mice. **A)** No genotype-dependent differences in body weight (genotype P=0.1711, $F_{1,36}$=1.951, DF=1). **B)** No genotype-dependent differences in gross neurologic function as composite score of gait, ledge balance, kyphosis, and hindlimb clasping (genotype P=0.5265, $F_{1,36}$=0.4091, DF=1). **C)** No genotype-dependent differences in grip strength normalized by body weight (genotype P=0.5745, $F_{1,36}$=0.3211, DF=1). **D)** No genotype-dependent differences in performance on rotorod task of motor coordination (genotype P=0.7647, $F_{1,7}$=0.0891, DF=1). **E)** No genotype-dependent differences in synaptic density per high-power field (HPF) in M1 (P=0.5395, $t_{1,16}$=0.627) or dorsal striatum (P=0.4693, $t_{1,16}$=0.7412). Statistics: A-D n=10 total animals per sex, per genotype; 2-way ANOVAs for factors genotype and sex; replicates=individual mice with error bars representing mean±SEM. E n=3 male 12wk old animals per genotype; unpaired *t*-test of group means; replicates=3 M1-dorsal striatum containing sections per animal with error bars representing mean±SEM.

Supplemental Figure 8 *Setd5*$^{+/-}$ animals fail to demonstrate elevated seizure activity by EEG. **A)** EEG is monitored for 16 hrs/day for 3 days. **B)** Ten-week-old *Setd5*$^{+/-}$ and littermates exhibit neither epileptic spikes nor seizure activities. **C)** To determine seizure threshold, a sub-convulsive dose of PTZ (20mg/kg) is injected (IP) was injected

into wild-type littermates and SETD5$^{+/-}$ mice (n=5, each) at 15 minutes intervals for 4 times until mice display seizure activities. **D)** Representative images show that gradual development of epileptic spikes and seizure activities in both *Setd5*$^{+/-}$ and littermates. **E)** Both *Setd5*$^{+/-}$ and littermates show increased epileptic spikes as PTZ dose increases (repeated measures two-way ANOVA, ($P<10^{-4}$, F(19.46)=64.47); however, no statistical difference is found between *Setd5*$^{+/-}$ and littermates (F(0.22) =0.2, P=0.6499). **F)** PTZ dose and time to induce seizure activities are not different between *Setd5*$^{+/-}$ and littermates ($t_{1,8}$=0.5373, P=0.6549, DF=8). Statistics: n=5 (wt) or n=3 (het) adult animals used as biological replicates (A-B) and n=5 each wt and het adult animals used as biological replicates (C-F), with ANOVA of group means compared at each timepoint post injection (E) and t-test of mean seizure threshold per animal (F).

Supplemental Figure 9 Absolute differences in brain region volume. Coronal series highlighting regional size differences with significance exceeding FDR (blue=smaller, red=larger). Statistics: n=12 (wt) or n=11 (het) adult brains as independent replicates.

Supplemental Figure 10 Significantly reduced thickness of CTIP2^{+} cortical neuron layer in *Setd5*$^{+/-}$ postnatal brain. **A-B)** Representative images of fluorescent P1 cortical sections, scale bar=50 µm. **C,E,G)** Immunohistochemical quantification of P1 cortical layers. At P1 no differences observed in thickness of TBR1^{+} layer (P=0.9090, $t_{1,56}$=0.1148, DF=56) or BRN2^{+} layer (P=0.5690, $t_{1,56}$=0.5729, DF=56), despite a significantly thinner CTIP2^{+} layer in *Setd5*$^{+/-}$ cortex (**P=0.0055, $t_{1,56}$=2.887, DF=56). At

P10 no differences observed in thickness of TBR1$^+$ layer (*P*=0.0945, $t_{1,43}$=1.71, DF=43) or BRN2$^+$ layer (*P*=0.0883, $t_{1,43}$=1.744, DF=43), despite a significantly thinner CTIP2$^+$ layer in *Setd5*$^{+/-}$ cortex (***P*=0.0012, $t_{1,43}$=3.466, DF=43). **D,F,H)** Cell density quantification, normalized to wild-type at each age. At P1, no difference in cell density within TBR1$^+$ layer (*P*=0.7481, $t_{1,55}$=0.3228, DF=55), CTIP2$^+$ layer (*P*=0.1064, $t_{1,55}$=1.642, DF=55), or BRN2$^+$ layer (*P*=0.5256, $t_{1,55}$=0.6388, DF=55). At P10, no difference in cell density within TBR1$^+$ layer (*P*=0.2191, $t_{1,42}$=1.248, DF=42), CTIP2$^+$ layer (*P*=0.4667, $t_{1,42}$=0.7345, DF=42), or BRN2$^+$ layer (*P*=0.1166, $t_{1,42}$=1.602, DF=42). Statistics: *n*=6 total animals per genotype, per age with *n*=2-3 images from distinct sections through M1 cortex as replicates, and *t*-test of group means. An individual replicate was considered the average of two separate measurements through the thickest and thinnest segments of the corresponding cell layer in a single image. Bars represent mean±SEM.

SUPPLEMENTAL REFERENCES

1. Beaudoin GM, 3rd, Lee SH, Singh D, Yuan Y, Ng YG, Reichardt LF*, et al*. Culturing pyramidal neurons from the early postnatal mouse hippocampus and cortex. *Nat Protoc* 2012; **7**(9)**:** 1741-1754.

2. Gompers AL, Su-Feher L, Ellegood J, Copping NA, Riyadh MA, Stradleigh TW*, et al*. Germline Chd8 haploinsufficiency alters brain development in mouse. *Nat Neurosci* 2017; **20**(8)**:** 1062-1073.

3. Chailangkarn T, Trujillo CA, Freitas BC, Hrvoj-Mihic B, Herai RH, Yu DX*, et al*. A human neurodevelopmental model for Williams syndrome. *Nature* 2016; **536**(7616)**:** 338-343.

4. Heinz S, Benner C, Spann N, Bertolino E, Lin YC, Laslo P*, et al*. Simple combinations of lineage-determining transcription factors prime cis-regulatory elements required for macrophage and B cell identities. *Mol Cell* 2010; **38**(4)**:** 576-589.

5. Li P, Spann NJ, Kaikkonen MU, Lu M, Oh DY, Fox JN*, et al*. NCoR repression of LXRs restricts macrophage biosynthesis of insulin-sensitizing omega 3 fatty acids. *Cell* 2013; **155**(1)**:** 200-214.

6. Tripathi S, Pohl MO, Zhou Y, Rodriguez-Frandsen A, Wang G, Stein DA*, et al*. Meta- and Orthogonal Integration of Influenza "OMICs" Data Defines a Role for UBR4 in Virus Budding. *Cell Host Microbe* 2015; **18**(6)**:** 723-735.

7. Zheng GX, Terry JM, Belgrader P, Ryvkin P, Bent ZW, Wilson R*, et al*. Massively parallel digital transcriptional profiling of single cells. *Nat Commun* 2017; **8:** 14049.

8. Satija R, Farrell JA, Gennert D, Schier AF, Regev A. Spatial reconstruction of single-cell gene expression data. *Nat Biotechnol* 2015; **33**(5)**:** 495-502.

9. Hinton LvdMaG. Visualizing Data using t-SNE. *Journal of Machine Learning* 2008; **9:** 2579-2605.

10. Osipovich AB, Gangula R, Vianna PG, Magnuson MA. Setd5 is essential for mammalian development and the co-transcriptional regulation of histone acetylation. *Development* 2016; **143**(24)**:** 4595-4607.

11. Guyenet SJ, Furrer SA, Damian VM, Baughan TD, La Spada AR, Garden GA. A simple composite phenotype scoring system for evaluating mouse models of cerebellar ataxia. *J Vis Exp* 2010;(39).

12. Tabuchi K, Blundell J, Etherton MR, Hammer RE, Liu X, Powell CM*, et al*. A neuroligin-3 mutation implicated in autism increases inhibitory synaptic transmission in mice. *Science* 2007; **318**(5847)**:** 71-76.

13. Katayama Y, Nishiyama M, Shoji H, Ohkawa Y, Kawamura A, Sato T*, et al*. CHD8 haploinsufficiency results in autistic-like phenotypes in mice. *Nature* 2016; **537**(7622)**:** 675-679.

14. Crawley JN. Mouse behavioral assays relevant to the symptoms of autism. *Brain Pathol* 2007; **17**(4)**:** 448-459.

15. Yang M, Silverman JL, Crawley JN. Automated three-chambered social approach task for mice. *Curr Protoc Neurosci* 2011; **Chapter 8:** Unit 8 26.

16. Bock NA, Nieman BJ, Bishop JB, Mark Henkelman R. In vivo multiple-mouse MRI at 7 Tesla. *Magn Reson Med* 2005; **54**(5)**:** 1311-1316.

17. Collins DL, Neelin P, Peters TM, Evans AC. Automatic 3D intersubject registration of MR volumetric data in standardized Talairach space. *J Comput Assist Tomogr* 1994; **18**(2)**:** 192-205.

18. Avants BB, Epstein CL, Grossman M, Gee JC. Symmetric diffeomorphic image registration with cross-correlation: evaluating automated labeling of elderly and neurodegenerative brain. *Med Image Anal* 2008; **12**(1)**:** 26-41.

19. Avants BB, Tustison NJ, Song G, Cook PA, Klein A, Gee JC. A reproducible evaluation of ANTs similarity metric performance in brain image registration. *Neuroimage* 2011; **54**(3)**:** 2033-2044.

20. Dorr AE, Lerch JP, Spring S, Kabani N, Henkelman RM. High resolution three-dimensional brain atlas using an average magnetic resonance image of 40 adult C57Bl/6J mice. *Neuroimage* 2008; **42**(1)**:** 60-69.

21. Nieman BJ, Flenniken AM, Adamson SL, Henkelman RM, Sled JG. Anatomical phenotyping in the brain and skull of a mutant mouse by magnetic resonance imaging and computed tomography. *Physiol Genomics* 2006; **24**(2)**:** 154-162.

22. Steadman PE, Ellegood J, Szulc KU, Turnbull DH, Joyner AL, Henkelman RM*, et al*. Genetic effects on cerebellar structure across mouse models of autism using a magnetic resonance imaging atlas. *Autism Res* 2014; **7**(1)**:** 124-137.

23. Ullmann JF, Watson C, Janke AL, Kurniawan ND, Reutens DC. A segmentation protocol and MRI atlas of the C57BL/6J mouse neocortex. *Neuroimage* 2013; **78:** 196-203.

24. Richards K, Watson C, Buckley RF, Kurniawan ND, Yang Z, Keller MD, *et al*. Segmentation of the mouse hippocampal formation in magnetic resonance images. *Neuroimage* 2011; **58**(3)**:** 732-740.

25. Genovese CR, Lazar NA, Nichols T. Thresholding of statistical maps in functional neuroimaging using the false discovery rate. *Neuroimage* 2002; **15**(4)**:** 870-878.

Fig S1

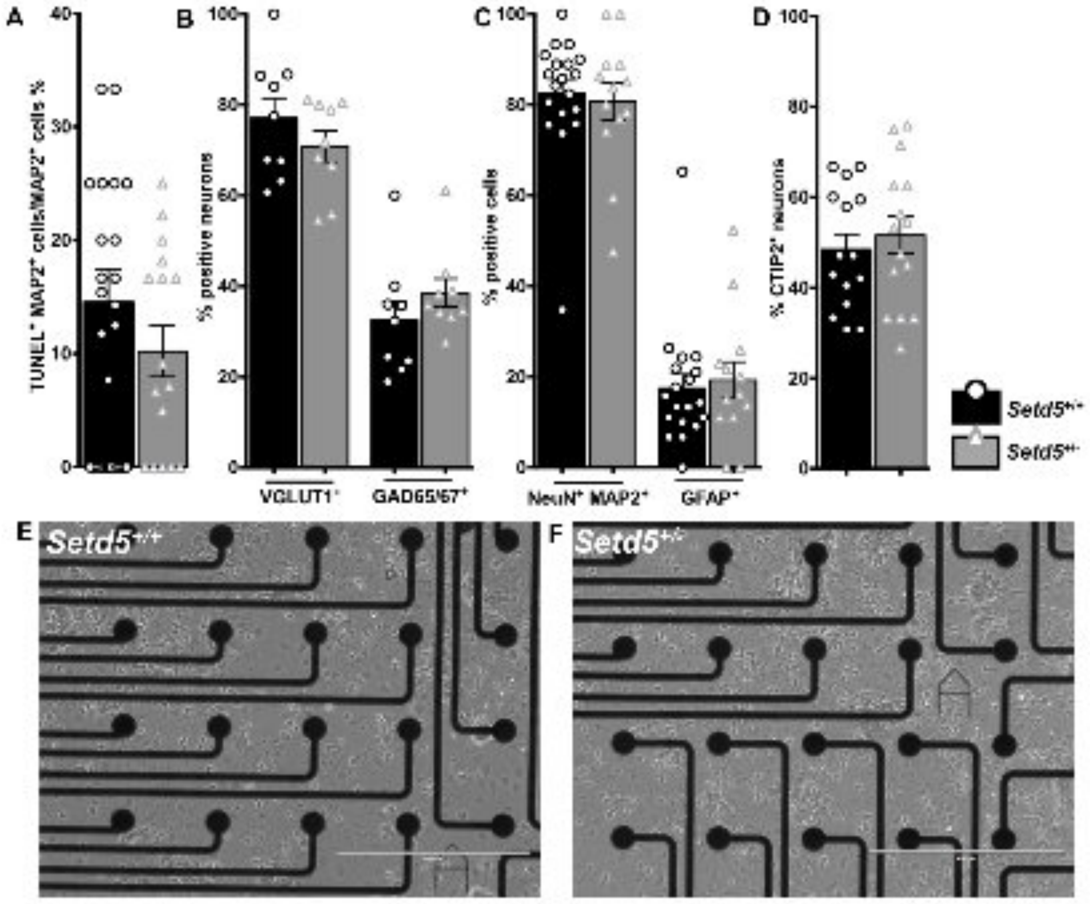
A
TUNEL+ MAP2+ cells/MAP2+ cells %
B
% positive neurons
VGLUT1+
GAD65/67+
C
% positive cells
NeuN+ MAP2+
GFAP+
D
% CTIP2+ neurons
Setd5+/+
Setd5+/-
E
Setd5+/+
F
Setd5+/-

Fig S2

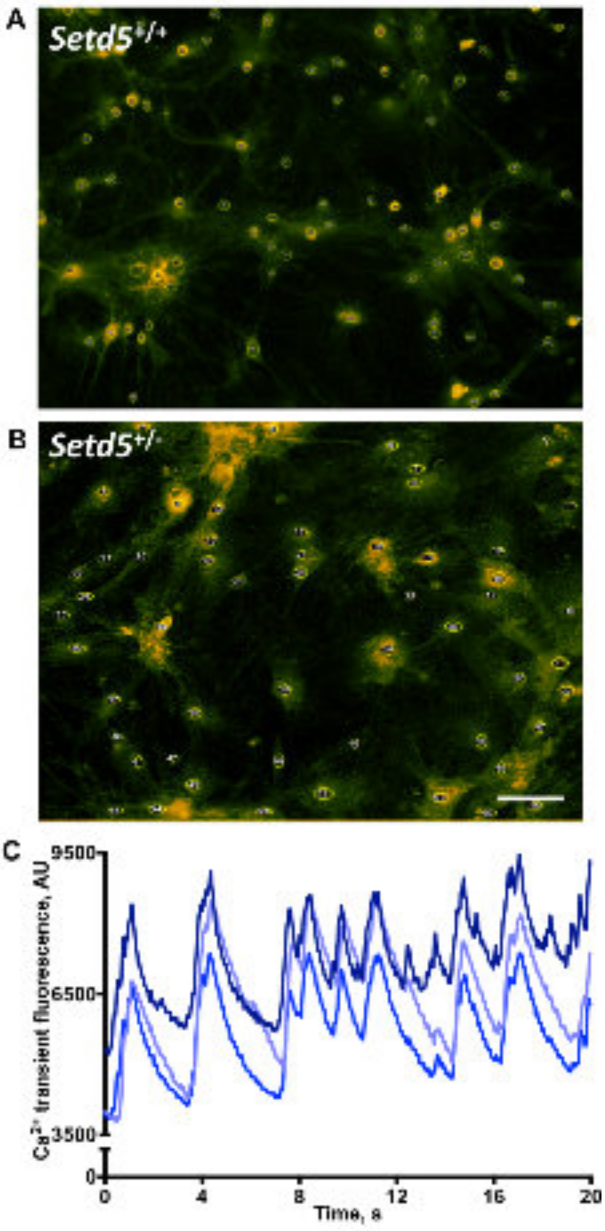

A
Setd5+/+
B
Setd5+/-
C
9500
6500
3500
0
0
4
8
12
16
20
Time, s
Ca2+ transient fluorescence, AU

Fig S3

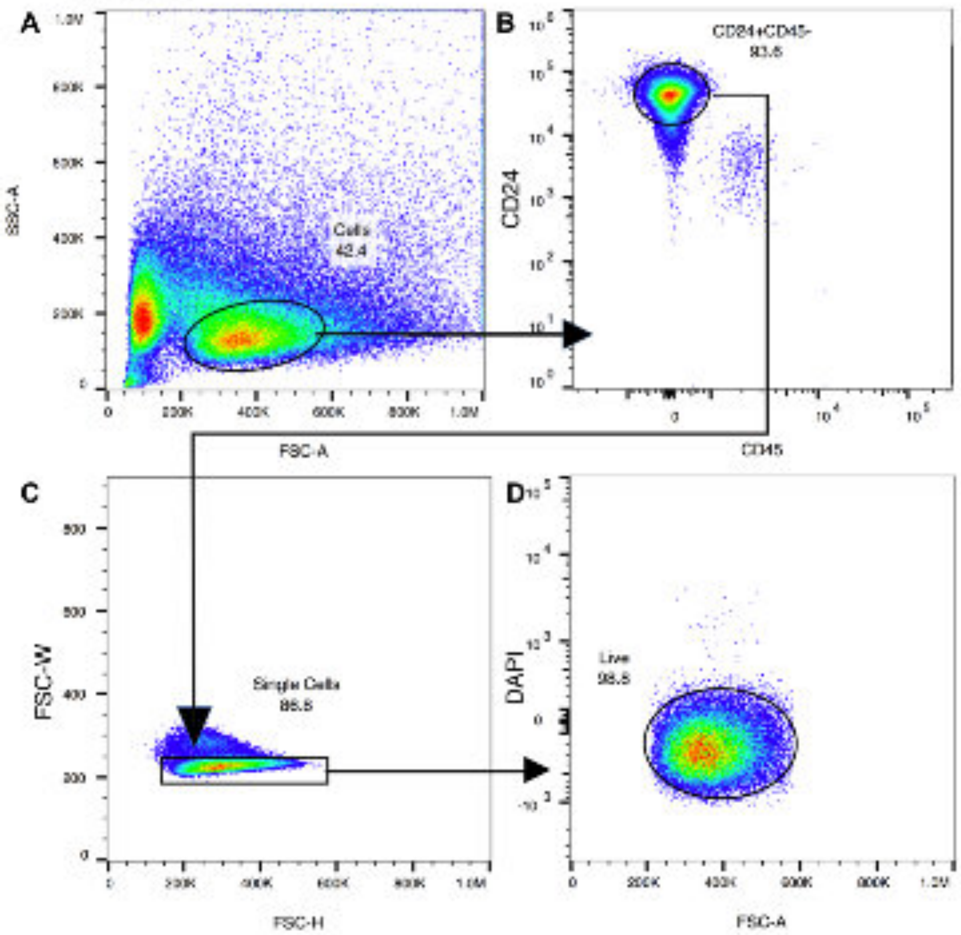
A
B
C
D
Cells
42.4
CD24+CD45-
93.6
Single Cells
Live
SSC-A
FSC-A
CD24
CD45
FSC-W
FSC-H
DAPI
FSC-A

Fig S4

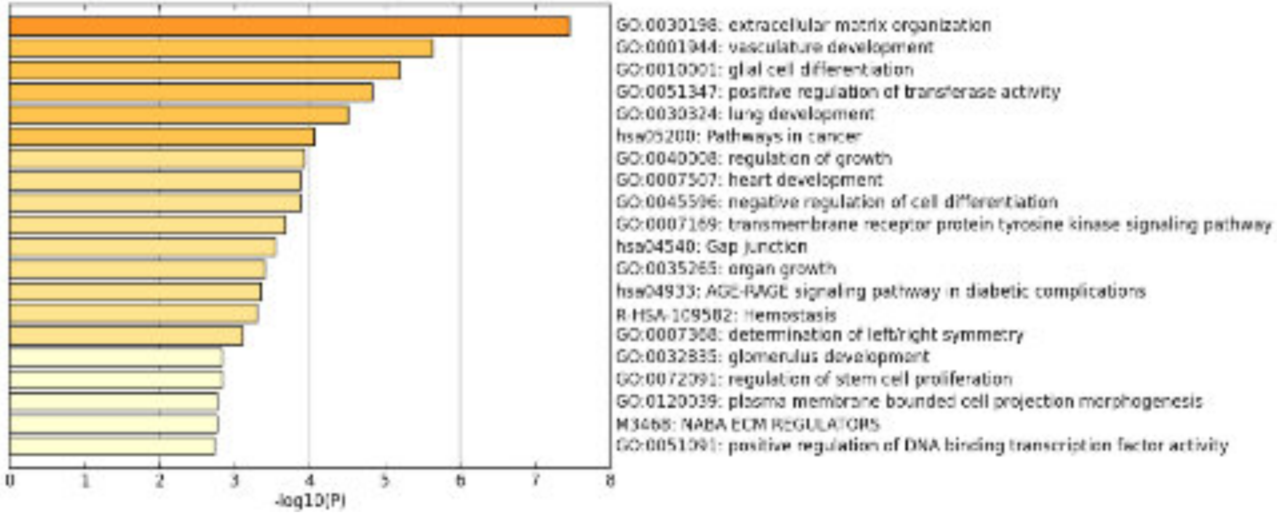
GO:0030198: extracellular matrix organization
GO:0001944: vasculature development
GO:0010001: glial cell differentiation
GO:0051347: positive regulation of transferase activity
GO:0030324: lung development
hsa05200: Pathways in cancer
GO:0040008: regulation of growth
GO:0007507: heart development
GO:0045596: negative regulation of cell differentiation
GO:0007169: transmembrane receptor protein tyrosine kinase signaling pathway
hsa04540: Gap junction
GO:0035265: organ growth
hsa04933: AGE-RAGE signaling pathway in diabetic complications
R-HSA-109582: Hemostasis
GO:0007368: determination of left/right symmetry
GO:0032835: glomerulus development
GO:0072091: regulation of stem cell proliferation
GO:0120039: plasma membrane bounded cell projection morphogenesis
M3468: NABA ECM REGULATORS
GO:0051091: positive regulation of DNA binding transcription factor activity
0
1
2
3
4
5
6
7
8
-log10(P)

Fig S5

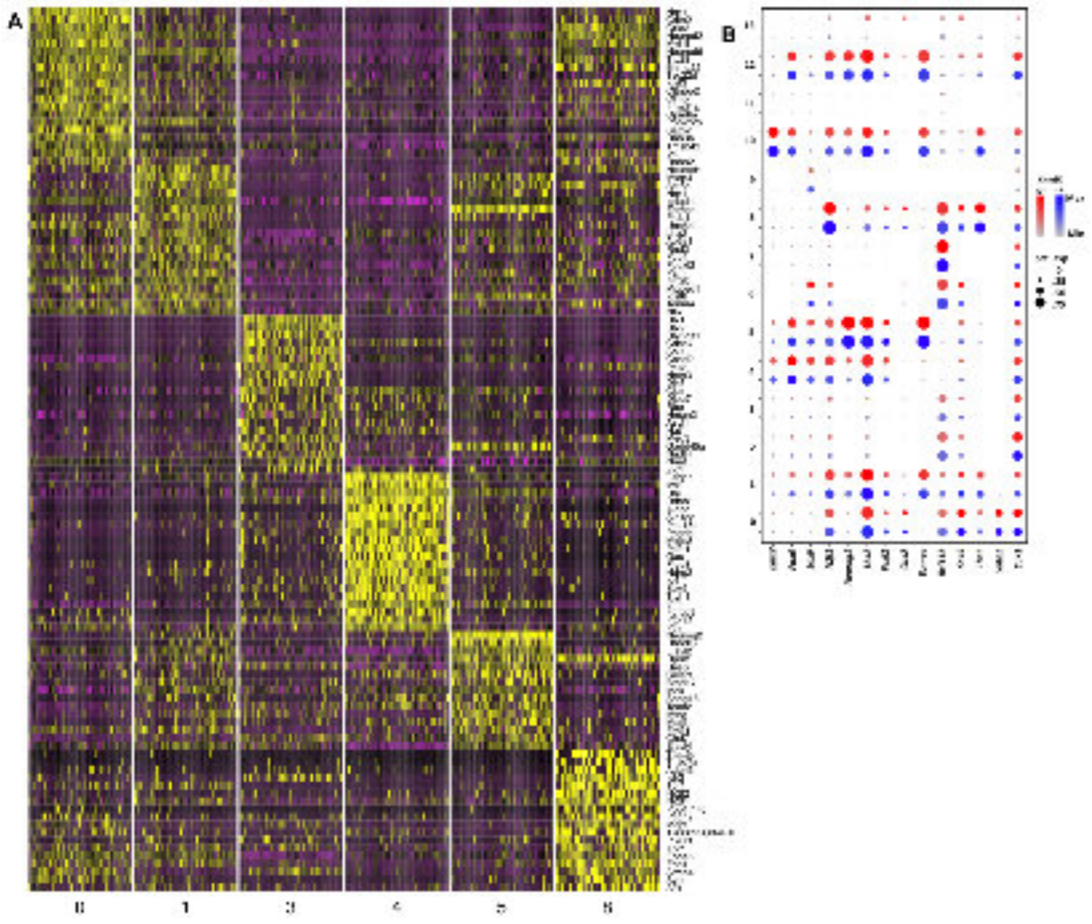

Fig S6

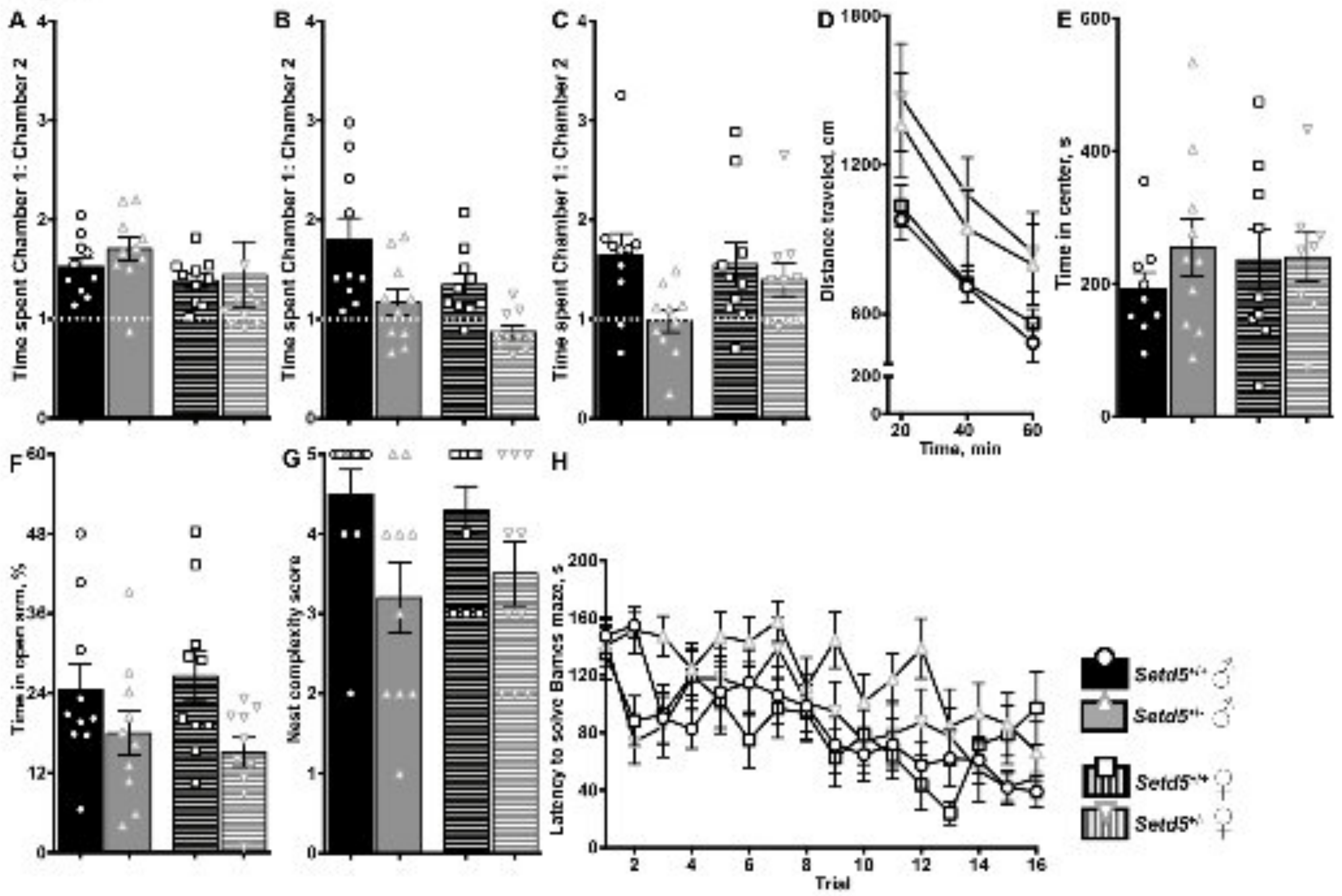

Fig S7

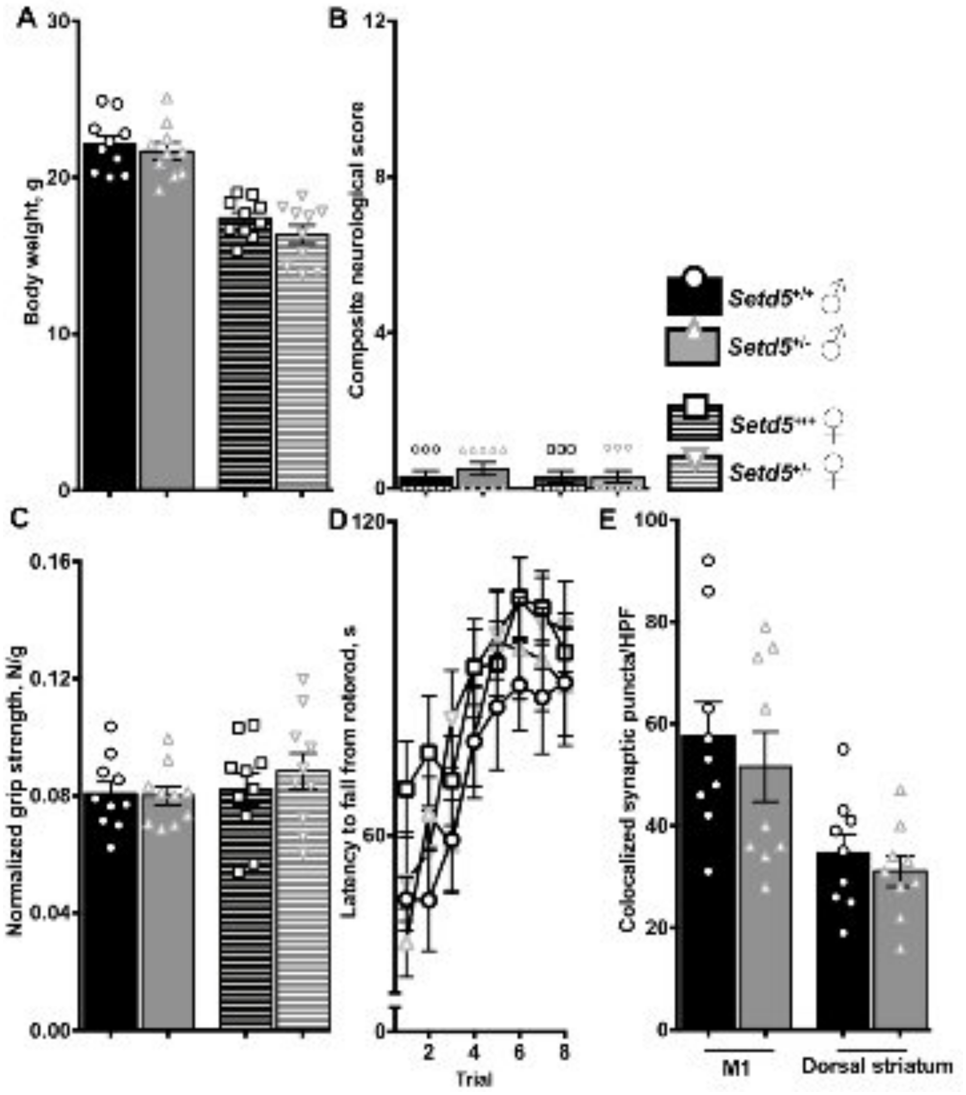
A
B
C
D
E
Body weight, g
Composite neurological score
Normalized grip strength, N/g
Latency to fall from rotorod, s
Trial
Colocalized synaptic puncta/HPF
M1
Dorsal striatum
Setd5+/+ ♂
Setd5+/- ♂
Setd5+/+ ♀
Setd5+/- ♀

Fig S8

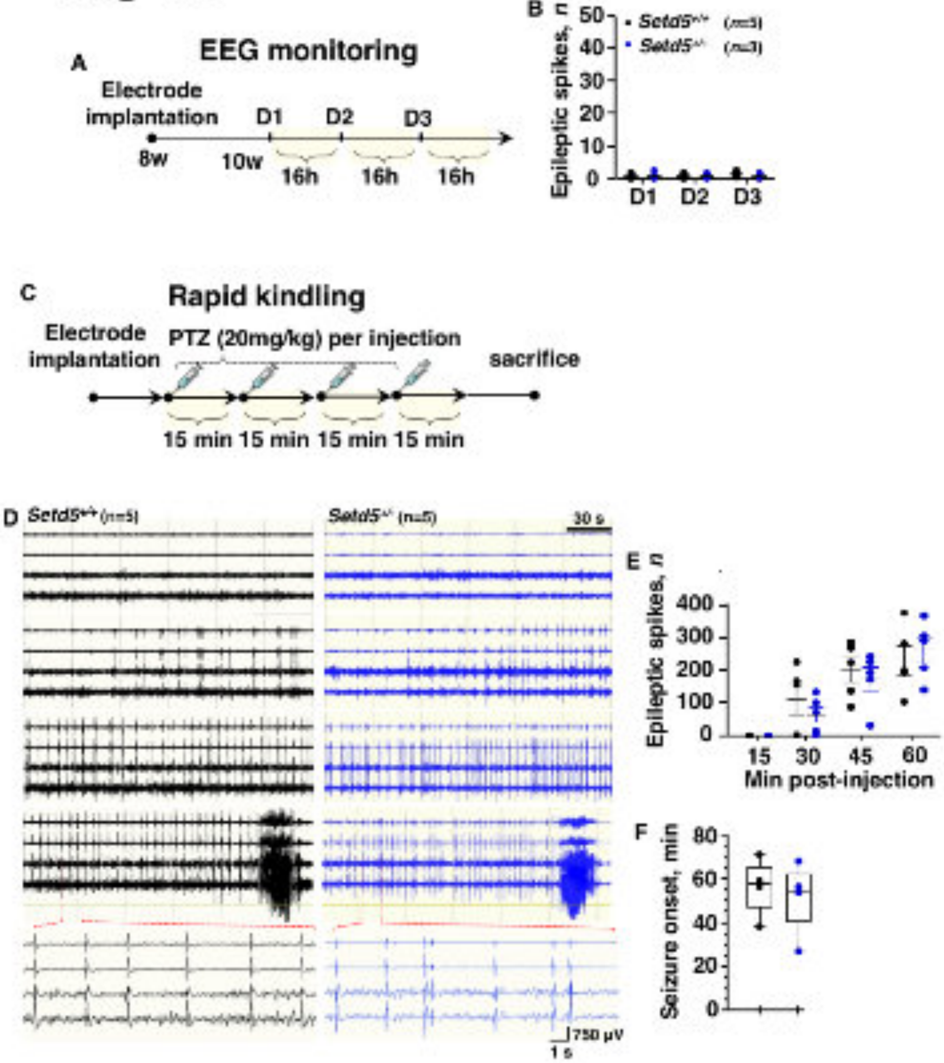
A
EEG monitoring
Electrode implantation
D1
D2
D3
8w
10w
16h
16h
16h
B
Setd5+/+ (n=5)
Setd5+/- (n=3)
Epileptic spikes, n
C
Rapid kindling
Electrode implantation
PTZ (20mg/kg) per injection
sacrifice
15 min 15 min 15 min 15 min
D
Setd5+/+ (n=5)
Setd5+/- (n=5)
30 s
750 µV
1 s
E
Epileptic spikes, n
Min post-injection
F
Seizure onset, min

Fig S9

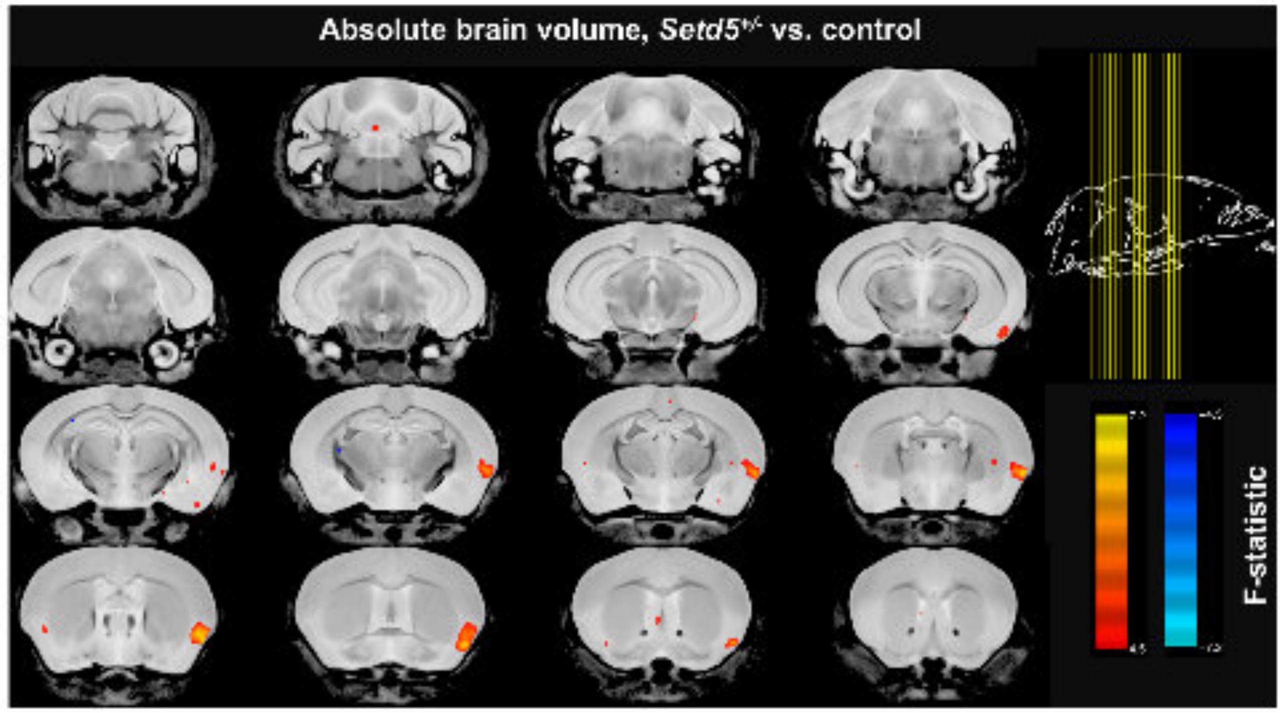

Fig S10

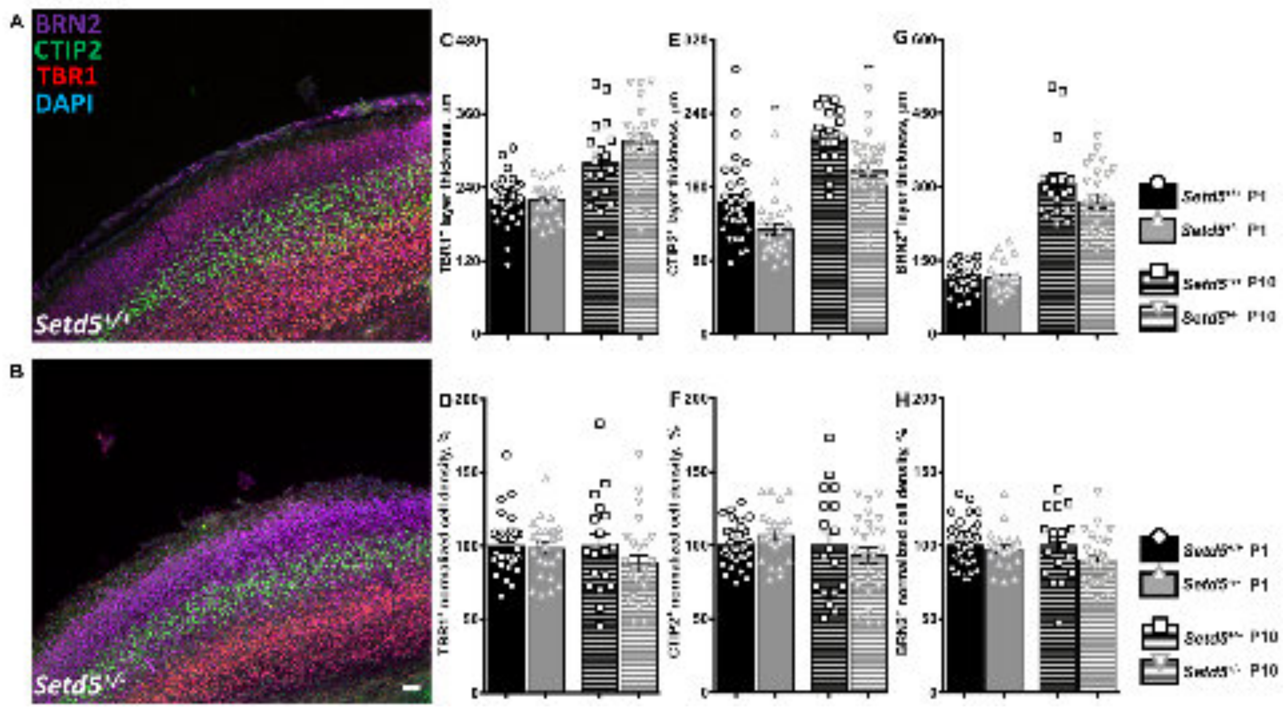

Supplement: Supplementary file 1 — Figure S1 [file 41398_2018_344_MOESM1_ESM.pdf]
